# The CRISPR‐Cas9 knockout *DDC* SH‐SY5Y *in vitro* model for AADC deficiency provides insight into the pathogenicity of R347Q and L353P variants: a cross‐sectional structural and functional analysis

**DOI:** 10.1111/febs.70120

**Published:** 2025-05-03

**Authors:** Cristian Andres Carmona‐Carmona, Giovanni Bisello, Rossella Franchini, Gianluigi Lunardi, Roberta Galavotti, Massimiliano Perduca, Rui P. Ribeiro, Benny Danilo Belviso, Alejandro Giorgetti, Rocco Caliandro, Patricia M.‐J. Lievens, Mariarita Bertoldi

**Affiliations:** ^1^ Department of Neurosciences, Biomedicine, and Movement Sciences University of Verona Italy; ^2^ Clinical Analysis Laboratory and Transfusional Medicine IRCCS‐Sacro Cuore Don Calabria Hospital Negrar Italy; ^3^ Department of Biotechnology University of Verona Italy; ^4^ Institute of Crystallography, CNR Bari Italy

**Keywords:** aromatic amino acid decarboxylase deficiency, CRISPR/Cas9, dopamine, knocked‐out *DDC* gene, neuroblastoma SH‐SY5Y cells

## Abstract

Aromatic amino acid decarboxylase (AADC) deficiency is a severe inherited recessive neurotransmitter disorder caused by an impairment in dopamine synthesis due to the lack/modification of AADC, the enzyme converting l‐dopa to dopamine. Patients exhibit severe movement disorders and neurodevelopmental delay, with a high risk of premature mortality. Given the lack of a reliable model for the disease, we developed a dopa decarboxylase knockout model using CRISPR/Cas9 technology in the SH‐SY5Y neuroblastoma cell line. This model showed a deficiency in AADC protein and activity, with an altered dopamine metabolites profile (low homovanillic acid and high 3‐O‐methyldopa) and a modified expression of key enzymes, such as dopamine beta‐hydroxylase and monoamine oxidases, which are involved in the catecholamine pathway. We then transfected the *DDC*‐KO cells with two AADC catalytic variants, R347Q and L353P, which resulted in loss‐of‐function and an altered profile of dopamine metabolites. By combining several structural approaches (X‐ray crystallography, molecular dynamics, small angle X‐ray scattering, dynamic light scattering, and spectroscopy), we determined that both variants alter the flexibility of the structural element to which they belong, whose integrity is essential for catalysis. This change causes a mispositioning of essential residues at the active site, leading, in turn, to an unproductive external aldimine, identifying the molecular basis for the loss‐of‐function. Overall, the *DDC*‐KO model recapitulates some key features of AADC deficiency, is useful to study the molecular basis of the disease, and represents an ideal system for small molecule screening regarding specific enzyme defects, paving the way for a precision therapeutic approach.

## Graphical abstract

Here, we generated a *DDC‐*KO cellular model for studying aromatic amino acid decarboxylase (AADC) deficiency. This model was created using CRISPR‐Cas9 in the SH‐SY5Y neuroblastoma cell line and was validated through the absence of the dopamine product and an altered l‐dopa metabolite profile. The combination of these results with additional structural and functional analyses obtained with purified recombinant wild‐type and Arg347Gln and Leu353Pro AADC deficiency variants provides insights into the molecular mechanisms of AADC pathogenic variants, which are crucial for the development of precision therapeutic approaches.

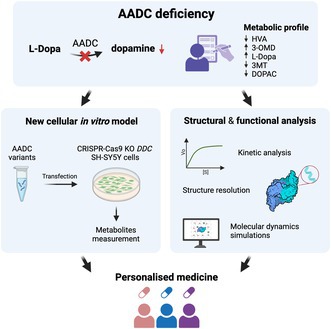

**Abbreviations**
AADC: aromatic amino acid decarboxylase
Ctrl: control
*DDC*
: dopa decarboxylase gene
KO: knocked‐out
MD: molecular dynamics
PLP: pyridoxal 5′‐phosphate
SAXS: small angle X‐ray scattering
SEC: size‐exclusion chromatography

## Introduction

The *dopa decarboxylase* (*DDC*) gene encodes aromatic l‐amino acid decarboxylase (AADC; EC4.1.1.28), the pyridoxal 5′‐phosphate (PLP)‐dependent enzyme responsible for the synthesis of dopamine and serotonin from l‐dopa and l‐5‐hydroxytryptophan, respectively. Biallelic mutations in this gene (7p12.2‐p12.1, chr7[hg38]: 50 458 436–50 565 460) cause a rare autosomal recessive disorder of the neurotransmitter metabolism: AADC deficiency. A high proportion of genotypes (90%) of the identified ~ 350 patients possess variants classified as pathogenic or likely pathogenic [1]. These patients are characterized by severe movement disorders and neurodevelopmental delay, with a high risk of premature mortality [2, 3, 4]. Given the recent impulse in diagnosis [5, 6, 7, 8, 9, 10, 11], by both screening for the 3‐O‐methyldopa marker and molecular genetic testing, the originally sporadic mild/moderate phenotypes now represent an emerging population of AADC deficiency patients [12] that would need to be addressed specifically and differently from the most affected ones.

After clinical evaluation, the diagnosis of the disorder is obtained by genetic testing to identify pathogenic/likely pathogenic variants on both alleles or by both cerebrospinal fluid (CSF)/plasma neurotransmitter profile consistent with AADC deficiency and a significantly reduced AADC activity in plasma [2, 3]. Pharmacologically targeted common treatments are pyridoxine, the precursor of PLP, monoamine oxidase inhibitors, and dopamine agonists associated with folinic acid and levodopa in selected cases [2]. Unfortunately, many patients do not respond satisfactorily to this pharmacological therapy [2].

A gene therapy treatment (Upstaza®) to putamen has been approved in Europe (2022)/the United Kingdom (2023) for the treatment of clinically severe patients (patients who cannot stand, walk, and sit) aged 18 months and older harboring a molecular, metabolic, and genetically confirmed diagnosis of AADC deficiency. The same treatment (named KEBILIDI®) has just been approved (November 2024) by the U.S. Food and Drug Administration and is authorized to be targeted to pediatric and adult patients with a diagnosis of AADC deficiency. Clinical studies of KEBILIDI did not include pediatric patients younger than 16 months or adult patients older than 65 years. A follow‐up study reporting the outcomes of this therapy is available only for a group of patients characterized by a common founder effect (an intronic mutation leading to a premature stop codon, geographically mainly localized in Taiwan and South Asia) [2] and could be considered partially satisfactory [13, 14, 15, 16], even if not resolutive. A different gene therapy treatment directed to the midbrain is under clinical trial, and the preliminary reports are encouraging [17]. Overall, alternative precision treatments to target severe and milder patients are required.

In order to study the molecular mechanisms of the disease, an appropriate model is necessary. Until now, there are few unsuitable models for the disease. *In vivo* mouse [18] or zebrafish [19] models show abnormal development but have poor survival and symptom relief in adults. Some *in vitro* cell models already used, such as Chinese Hamster Ovarian (CHO) cells, are inappropriate since they do not express AADC and cannot be suitable for illustrating dopamine metabolism [20]. The best *in vitro* models are patient‐derived cell models [21] that represent a good platform to develop and investigate precision therapeutic approaches but are expensive and require efficient reprogramming and extensive validation. Thus, a suitable model to study the disease's molecular mechanisms underlying different AADC variants is urgent as it could be beneficial to unravel the molecular basis for the individual defect and model therapeutic alternative treatments. Indeed, the neuroblastoma SH‐SY5Y cell line could represent a proper model given its neuronal origin and the ability to express AADC and the enzymes of the dopamine metabolism pathway [22, 23].

In this study, we knocked‐out (KO) *DDC* in SH‐SY5Y cells and evaluated its ability as an *in vitro* model for AADC deficiency by determining that both AADC protein amount and activity are absent. Then, we measured the levels of dopa metabolites, which overlap with the metabolites profile shown by AADC deficiency patients. As a further step, we transfected the KO‐*DDC* cell model with wild‐type (WT) AADC and two highly affected catalytic variants [24, 25, 26], R347Q and L353P, and evaluated protein amount, AADC activity, and metabolite profile in all cases. These results were associated with the first crystallographic structures of AADC variants, R347Q and L353P, coupled with molecular dynamics (MD) simulations and small angle X‐ray scattering (SAXS) analyses to define the molecular cause of the defects. The combination of results obtained from the *DDC*‐KO cell model associated with the structural features determined from the purified recombinant variants is crucial to the comprehension of the molecular event that leads to an impaired AADC species. This is an approach applied for the first time to AADC deficiency and could pave the way to the design of appropriate small molecules for possible alternative treatment of the disorder, especially in patients not addressed to gene therapy.

## Results

### Generation of *DDC* knockout clones in SH‐SY5Y cells by CRISPR/Cas9 and cytotoxic effect of l‐dopa

**Fig. 1:**
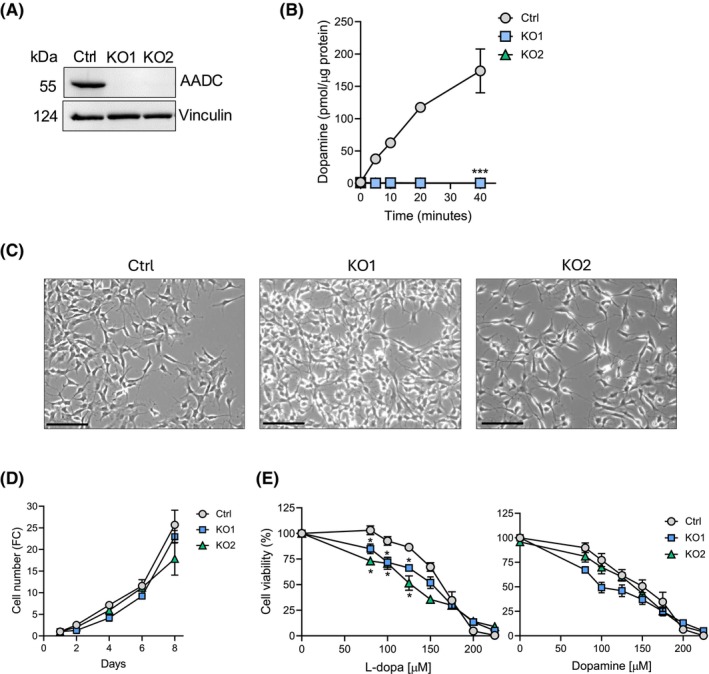
AADC expression and activity in *DDC*‐KO clones and cytotoxic effects of l‐dopa. (A) Western blot for AADC expression of KO1 and KO2 clones. Vinculin was used as a loading control. A representative blot is shown (*n* = 3). (B) AADC activity assay over time relative to the total protein. Data are represented as mean ± SEM of three independent experiments. ***P < 0.001 in comparison to the neuroblastoma SH‐SY5Y parental cells (control, ctrl). (C) Bright‐field microscopy images of WT and *DDC*‐KO cells. Scale bar: 50 mm. (D) Proliferation of control and *DDC*‐KO cells counted by Trypan blue exclusion assay. Values are reported as fold change (FC) relative to 1 day of incubation. Data are represented as mean ± SEM of three independent experiments. No significant differences were observed between ctrl and DDC‐KO cells (*n* = 3, one‐way ANOVA followed by Dunnett's test). (E) Cell viability of WT and *DDC*‐KO cells after treatment with l‐dopa and dopamine for 24 h. Data are represented as mean ± SEM of three independent experiments. *P < 0.05 in comparison to the control cells (*n* = 3, one‐way ANOVA followed by Dunnett's test).

To knock out the human *DDC* gene in the neuroblastoma SH‐SY5Y cell line, we used the CRISPR/Cas9 system (Fig. S1A) via non‐homology base repair. Given the trisomy of chromosome 7 (where *DDC* is located) in the SH‐SY5Y cell line [27], we carried out a deep characterization in the process of clone selection (identification of the genetic modifications determined by DNA sequencing of the three alleles, qPCR, western blot, and AADC activity assays) whose details are shown (Fig. S1B–E). After selecting puromycin‐resistant clones, western blotting was performed to screen the expression of AADC protein (Fig. 1A). This screening revealed two clones with a null expression of AADC (henceforth named *DDC*‐KO1 and *DDC*‐KO2 with respect to the neuroblastoma SH‐SY5Y control cells). Consequently, these clones showed a complete absence of AADC enzymatic activity (Fig. 1B). Altogether, our results indicated that *DDC*‐KO clones are derived from single cells, in which each clone harbors the same set of total loss‐of‐function mutations in the *DDC* gene. The absence of AADC protein was permanent after different passages, allowing long‐term analysis of these modified cell lines.

The human neuroblastoma cell line SH‐SY5Y is characterized by the presence of neuroblastic cells (N‐type) and substrate adherent cells (S‐type) [28]. With respect to the parental SH‐SY5Y neuroblastoma cell line, *DDC*‐KO clones presented mainly neuroblastic‐type cells, and the population remained invariable over the passages (Fig. 1C). To determine the relevance of AADC for cell growth in basal conditions, we assessed the proliferation rate of control and *DDC*‐KO clones upon 2–8 days of culture in a complete medium. *DDC*‐KO cells grew normally compared to control cells under standard cultured conditions (Fig. 1D). Subsequently, these cells were treated with l‐dopa and dopamine at different concentrations for 24 h, and cell viability was evaluated by OZ Blue test (Fig. 1E). We observed that *DDC‐*KO clones were more sensitive to l‐dopa than control cells at low concentrations of l‐dopa, while concentrations over 150 μm were equally toxic for control and *DDC*‐KO cells. In contrast, the cytotoxic effect of dopamine was similar between control and *DDC*‐KO clones. These results demonstrate that the absence of AADC in SH‐SY5Y KO cells does not affect their proliferation but makes them more sensitive to the cytotoxic effects of l‐dopa.

### *DDC*‐KO modulates the expression of enzymes involved in dopamine metabolism

**Fig. 2:**
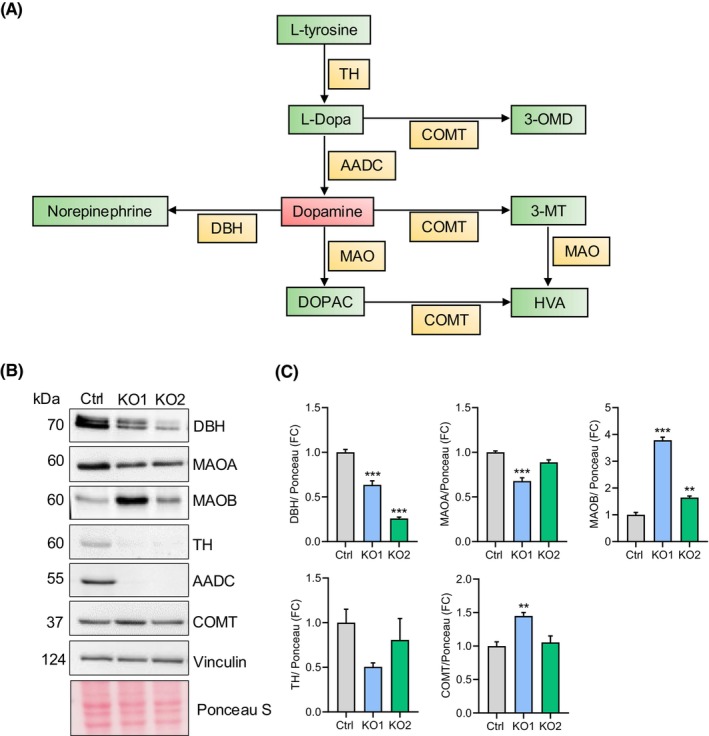
The expression of enzymes involved in dopamine metabolism pathway in *DDC*‐KO and control SH‐SY5Y cells is differently modulated. (A) Scheme of the dopaminergic pathway showing the enzymes (in yellow) involved in each step and the metabolites (in green). Dopamine is in red as it represents the impaired point. 3MT, 3‐methoxy tyramine; 3‐OMD, 3‐O‐methyldopa; AADC, aromatic amino acid decarboxylase; COMT, catechol‐O‐methyl transferase; DBH, dopamine beta‐hydroxylase; DOPAC, 3,4‐dihydroxyphenylacetic acid; HVA, homovanillic acid; MAO, monoamine oxidase; TH, tyrosine hydroxylase. (B) Representative western blot of the main enzymes related to the biosynthesis and clearance of dopamine in control cells and *DDC* knockout clones (KO1 and KO2). Ponceau S and Vinculin are shown as loading controls (*n* = 3). (C) Densitometry analysis of three independent experiments. Values are reported as fold change (FC) relative to the control cells. Bars represent the mean ± SEM. Significant differences were determined with one‐way ANOVA followed by Dunnett's test. **P < 0.01 and ***P < 0.001 in comparison to the control cells.

To determine whether removing AADC could modify the expression of other enzymes involved in dopamine metabolism (Fig. 2A), we evaluated the expression of tyrosine hydroxylase (TH), dopamine beta‐hydroxylase (DBH), catechol‐O‐methyltransferase (COMT) and monoamine oxidases A and B (MAOA and MAOB) by western blot in basal conditions (Fig. 2B). Interestingly, the control and *DDC*‐KO cells showed barely detectable levels of TH, and the bands in the western blot were only detected after long time exposures. The intensity of these bands was variable according to the passage number, becoming more evident in the first passages (data not shown). The densitometry analysis did not reveal a statistical difference in the TH expression between control and *DDC*‐KO cells (Fig. 2C). DBH expression was reduced in *DDC*‐KO clones compared to control cells (Fig. 2B,C). Conversely, COMT expression was higher in *DDC*‐KO clones than in control cells; this difference was more significant in the *DDC*‐KO1 (Fig. 2B,C). By comparing the expression levels of MAOA and MAOB, we found that they were regulated in opposite directions in *DDC*‐KO clones: MAOA was decreased, while MAOB was markedly increased compared to control cells (Fig. 2B,C). These findings indicate that knocking out *DDC* in SH‐SY5Y cells alters the expression of proteins implicated in dopamine metabolism.

### Transient expression of WT AADC and R347Q and L353P pathogenic variants in *DDC*‐KO SH‐SY5Y cells

**Fig. 3:**
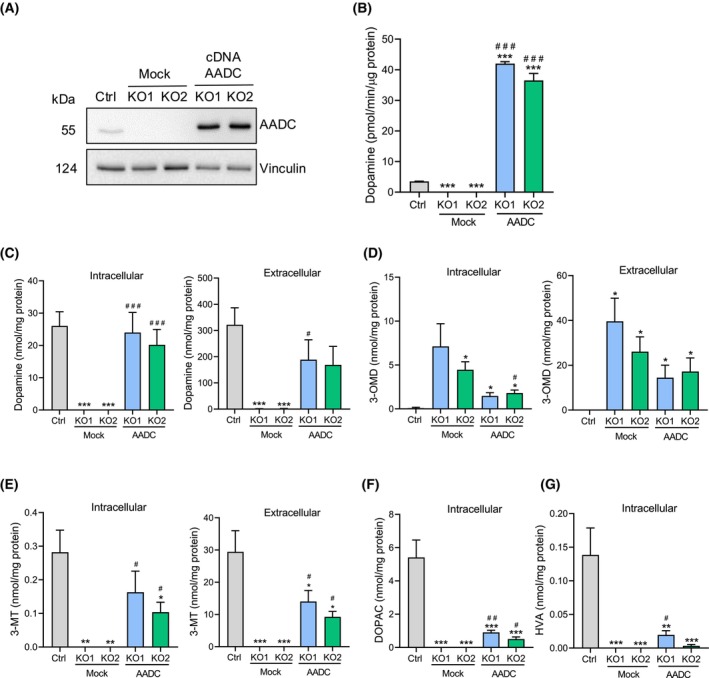
*DDC*‐KO cells exhibit altered levels of dopamine and its downstream metabolites. (A) Western blot of AADC levels from *DDC*‐KO cells transiently overexpressing the AADC wild‐type. Transfected *DDC*‐KO cells with a reaction that lacks DNA were used as negative control (mock). A representative blot is shown (*n* = 3). (B) AADC activity assay relative to total protein. Data are represented as mean ± SEM of three independent experiments. (C–G) Detection of intracellular and extracellular dopamine and its metabolites by mass spectrometry. 3MT, 3‐methoxy tyramine; 3‐OMD, 3‐O‐methyldopa; DOPAC, 3,4‐dihydroxyphenylacetic acid; HVA, homovanillic acid. Cells were treated with 80 μm l‐dopa for 2 h. Values are normalized to the total protein. Data are represented as mean ± SEM of four independent experiments. Significant differences were determined with one‐way ANOVA followed by Dunnett's test. Statistical legends: *P < 0.05, **P < 0.01 and ***P < 0.001 in comparison to the control cells. ^#^ *P* < 0.05, ^##^ *P* < 0.01 and ^###^ *P* < 0.001 in comparison to the respective mock cells.

When *DDC*‐KO cells were transfected with a pCMV‐*DDC* expression vector, a clear band due to AADC protein expression appeared (Fig. 3A), and AADC activity was restored (Fig. 3B). It is known that the absence of functional AADC leads to altered levels of dopamine in plasma and cerebrospinal fluid (CSF) of AADC deficiency patients [2], who in addition present also low dopamine catabolite levels (such as 3‐methoxy tyramine (3‐MT), 3,4‐dihydroxyphenylacetic acid (DOPAC), and homovanillic acid (HVA)) and high amounts of the l‐dopa derivative 3‐O‐methyldopa (3‐OMD), suggested to be supportive as a biomarker for AADC deficiency [3, 5]. To evaluate whether these alterations are present in the *DDC*‐KO clones, we measured the intracellular and extracellular concentrations of dopamine and its downstream metabolites by mass spectrometry. Notably, these metabolites were not detectable in control or *DDC*‐KO clones in basal conditions independently of the number of cells (data not shown), as expected [29]. Therefore, cells were treated with 80 μm l‐dopa for 2 h in a media supplemented with 1% FBS. Upon l‐dopa treatment, the metabolites dopamine, 3‐MT, DOPAC, and HVA were only found in control cells (Fig. 3C,E–G). The last two metabolites were present merely in the intracellular samples. In contrast, 3‐OMD was the only metabolite in the *DDC*‐KO samples (Fig. 3D). To confirm the specificity of this metabolic phenotype induced by loss of AADC, we performed rescue experiments using a pCMV‐*DDC* expression vector. The re‐expression of AADC restored the production of dopamine in *DDC*‐KO clones (Fig. 3C). Overexpression of AADC in *DDC*‐KO cells also led to the biosynthesis of 3‐MT, DOPAC, and HVA, but the levels of these metabolites were substantially reduced compared to the control cells (Fig. 3E–G). The concentration of 3‐OMD was also decreased without achieving the levels of the control cells (Fig. 3D). The levels of these metabolites were similar between *DDC*‐KO1 and *DDC*‐KO2, indicating that the l‐dopa effect on *DDC*‐KO cells is not clone‐dependent.

To determine whether *DDC*‐KO SH‐SY5Y cells could model the activity effects and metabolites spectrum of AADC pathogenic variants, we transiently expressed the R347Q and L353P variants in parallel with the WT in the selected *DDC*‐KO1 clone. R347Q is the most frequent missense variant causing AADC deficiency (allelic frequency = 6.9%), and L353P is moderately frequent (allelic frequency = 0.6%) [1]. Moreover, both of them deeply affect catalysis [24, 25, 26] since they belong to a structural element essential for the correct catalytic activity of AADC [30].

**Fig. 4:**
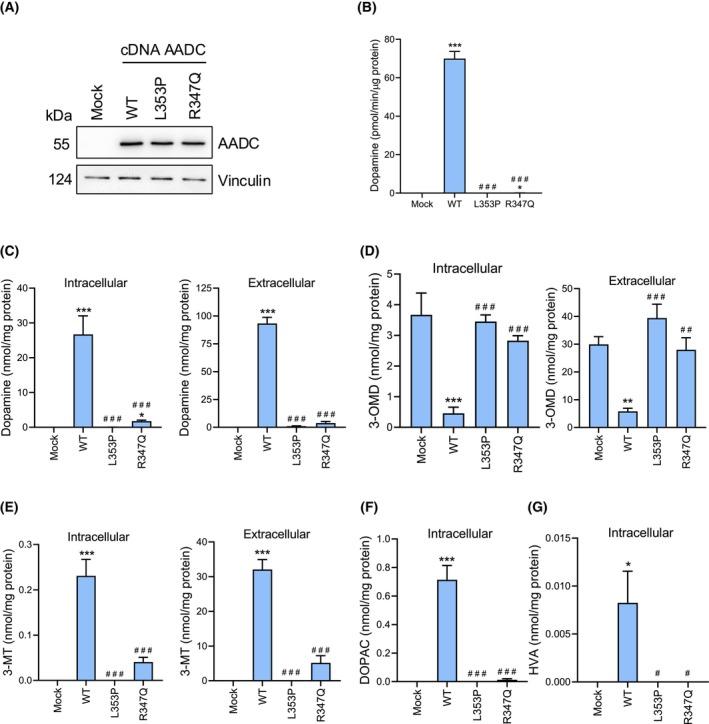
Determination of expression levels, enzymatic activity and dopamine metabolites levels *DDC*‐KO clone transfected with R347Q and L353P AADC variants. (A) Western blot of AADC levels from *DDC*‐KO1 cells transiently overexpressing the WT AADC or the pathogenic R347Q and L353P variants. Transfected *DDC*‐KO1 cells with a reaction that lacks DNA were used as negative control (mock). A representative blot is shown (*n* = 3). (B) AADC activity assay relative to total protein. Data are presented as mean ± SEM of three independent experiments. (C–G) Detection of intracellular and extracellular dopamine and its metabolites by mass spectrometry. 3MT, 3‐methoxy tyramine; 3‐OMD, 3‐O‐methyldopa; DOPAC, 3,4‐dihydroxyphenylacetic acid; HVA, homovanillic acid. Cells were treated with 80 μm l‐dopa for 2 h. Values are normalized to the total protein. Data are represented as mean ± SEM of four independent experiments. Significant differences were determined with one‐way ANOVA followed by Dunnett's test. Statistical legends: *P < 0.05, **P < 0.01 and ***P < 0.001 in comparison to mock cells. ^#^ *P* < 0.05, ^##^ *P* < 0.01 and ^###^ *P* < 0.001 in comparison to the cells overexpressing the AADC wild‐type.

We first determined that protein expression is comparable for cells transfected with both pathogenic variants and WT protein, inferring similar intracellular protein stability, as suggested by studies with the recombinant species [24, 25, 26] (Fig. 4A). AADC activity was significantly lower or absent in cells expressing the two catalytic variants with respect to the WT, as expected (Fig. 4B). The impaired catalytic activity was also reflected in the biosynthesis of dopamine (Fig. 4C) and its downstream metabolites upon the l‐dopa challenge. In particular, 3‐OMD is increased, while 3‐MT, DOPAC, and HVA are decreased compared to WT expression (Fig. 4D–G).

Overall, the results indicate that *DDC*‐KO SH‐SY5Y cells treated with l‐dopa are suitable to mimic the AADC variants' effects in terms of protein expression, enzymatic function, and metabolite levels. In addition, the catalytic R347Q and L353P variants do not synthesize dopamine and determine a metabolic profile typical of an impaired l‐dopa pathway. The molecular cause for the loss of function of these catalytic variants has been thus investigated by high‐ and low‐resolution static and dynamic structural techniques.

### The crystal structures of R347Q and L353P AADC variants present subtle changes in the loop3 segment

Recombinant R347Q and L353P AADC variants were expressed, purified, and characterized in more detail than previously published [24, 26], and collectively, they maintain an unaltered secondary structure (Fig. S2A), a comparable thermal stability (Fig. S2B), a similar tertiary structure as well as the PLP microenvironment with respect to the WT (Fig. S2C,D). However, slight anomalies exist in the spectral signal of the external aldimine with the l‐dopa analogue l‐dopa methylester (DME) (Fig. S2E), which mimics the productive catalytic complex. Thus, it may be suggested that the basis for the almost total loss‐of‐function of these variants could be due to subtle active site effects that prevent the correct orientation of the catalytically competent external aldimine species [31, 32]. Since the residues Arg347 and Leu353 belong to a region (loop3) dynamically associated with the catalytic capability [30], we solved the crystal structures of the two catalytic variants and coupled them to MD simulations.

The crystal structures of R347Q and L353P were solved as a monomer in the asymmetric unit (Space group P6_1_22) at 1.7 Å (PDB ID: 9HRH) and 2.05 Å (PDB ID: 9HRI), respectively (Table S1).

**Fig. 5:**
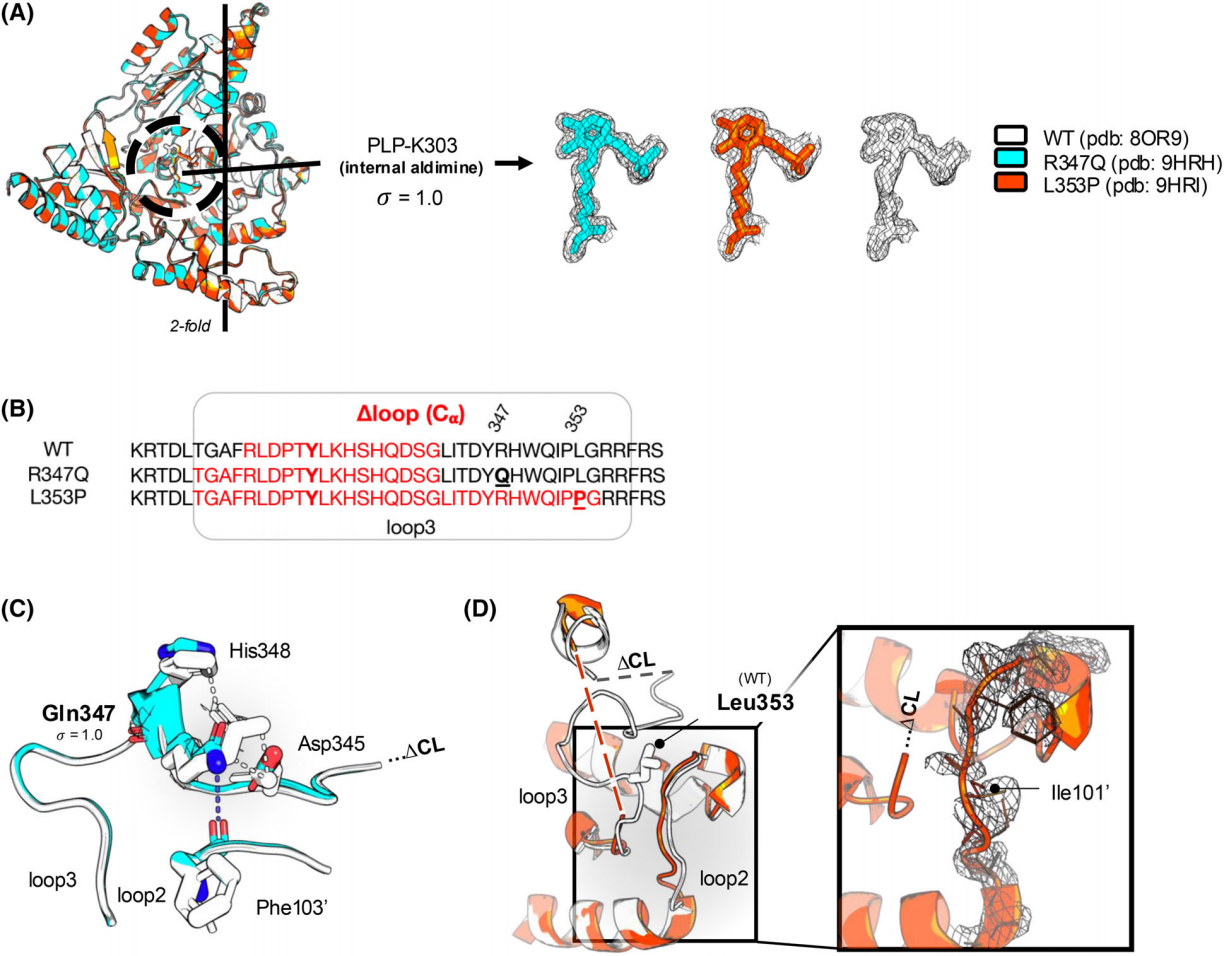
Crystal structure of R347Q and L353P AADC variants. (A) Superposition of monomeric R347Q (cyan) and L353P (red) variants on native WT AADC (white). The 2‐fold axis is indicated, and the active site region is circled. The PLP‐Lys303 internal aldimine electron density of WT and of the variants is contoured at 1.0σ. (B) Amino acid sequence alignment comparing the length of missing electron density stretches (∆loop, in red) of WT, R347Q, and L353P crystal structures. Protein accession number for AADC is UniProt P20711‐1. Amino acids belong to the same sequence and are manually aligned in the loop3 region. Loop3 (residues 324–357) is circled in gray and residues 347 and 353 (substituted in the respective variants) are bold and underlined. (C) Superposition of R347Q structure (cyan) to native WT (white), showing the interactions between Arg347 and Asp345 and His348 belonging to loop3, and the new interaction made by Gln347 (with its electron density contoured at 1.0σ) and Phe103 of adjacent loop2. (D) L353P structure (red) superposed to the native WT (white) showing that the entire loop3 is missing. In the inset, L353P loop2 electron density at 1.0σ. Images were rendered with pymol 2.3.4 (Schrödinger Inc, New York, NY, USA).

Overall, they show high similarity with the WT (root mean square deviation (RMSD) ≅ 0.092 Å for R347Q and ≅ 0.140 Å for L353P) (Fig. 5A left), and the PLP‐Lys303 internal aldimine shows analogous electron density as the WT (Fig. 5A right). Even if the active site of the two variants does not show relevant differences in comparison to the WT structure, a main difference among the three structures is however amenable to the flexibility of the loop3 region (residues 324–357). WT AADC internal aldimine structure (PDB ID: 8OR9) [30] is missing the highly flexible catalytic loop (CL, residues 327–341) containing the catalytic residue Tyr332 [33]. Similarly, the electron density in this region is missing in the two variants. Still, for a longer stretch: 19 residues (323–341) for R347Q and 32 residues (323–354, also containing the residues at position 353) for L353P (Fig. 5B). In more detail, while Arg347 in WT AADC is involved in the stabilization of loop3 by contacting Asp345 and His348 [30], in the crystal structure of R347Q, despite the poor electron density, Gln347 is oriented toward the backbone of Phe103′ of loop2, determining a slight tilt of Asp345 and His348 with respect to the WT structure (Fig. 5C). Leu353, at the C‐terminal part of loop3, concurs in the stabilization of the PLP phosphate group [30]. In the crystal structure of L353P the electron density of almost the entire loop3 is missing, including Pro353, indicating overall increased flexibility of this region (Fig. 5D). Moreover, loop2 of the L353P structure shows a scarce electron density (i.e., Ile101′, a substrate stabilizing residue [30] is invisible) as well as a different backbone conformation to the WT.

Overall, crystallographic data of the two catalytic variants confirm subtle changes at the active site and higher flexibility of the CL segment. Since the structural dynamics of the CL are coupled to enzyme function [30], we wondered if the defect of these catalytic variants is due to the conformational mobility of the CL and thus carried out all‐atom MD simulations.

### All‐atom MD simulations of R347Q and L353P AADC variants show that these variants are impaired in a catalytically crucial flexible region

**Fig. 6:**
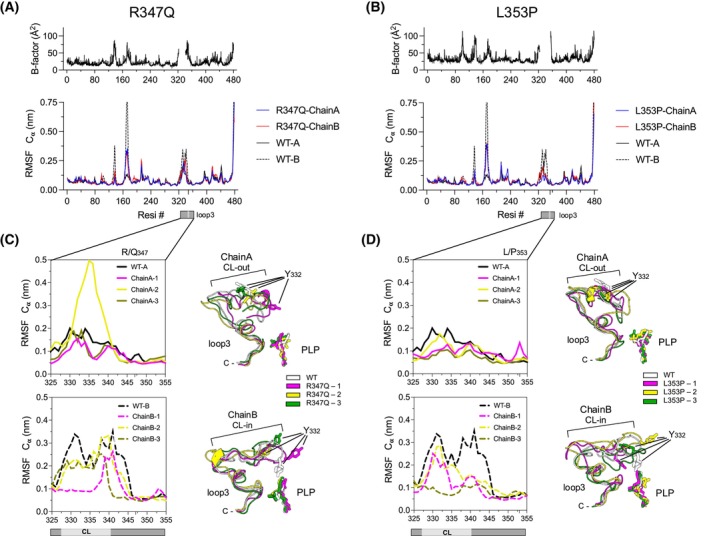
RMSF profile and loop3 flexibility of R347Q and L353P variants in all‐atom molecular dynamics simulations. (A, B) RMSF profile and crystallographic B‐factors of R347Q and L353P variants, respectively. Chain A is represented with a solid blue line while Chain B with a solid red line. The RMSF of WT AADC [30] (chain A solid black line and chain B dashed black line) is reported for reference. The RMSF values for the variants are obtained from the average of three replicas and the shaded area represents the SD. The Loop3 region is highlighted by a gray box with the substitution position shown in light gray; (C, D) zoom of RMSF loop3 in R347Q and L353P simulations, respectively. Representative clusters of each MD trajectory of CL‐out and CL‐in conformations are shown in different colors on the right. The PLP‐Lys303 internal aldimine and the catalytic Tyr332 are labeled. CL, catalytic loop. Images were rendered with pymol 2.3.4 (Schrödinger Inc).

The measurement of the root mean square fluctuation (RMSF) shows that the flexibility of the Ca of both chains of the two variants along the sequence is in line with the experimental B‐factor and is similar to that of the WT (Fig. 6A,B). The RMSD and the radius of gyration of R347Q and L353P indicate that the substitutions do not affect the global dimer size and shape (Fig. S3A,B and Table S2).

The most flexible regions, characterized by high B‐factor values, overlap with those identified in the WT [30]. Differently from the WT, the variants show a reduced flexibility in the short loop around amino acid 136 and a reduced asymmetric flexibility between chain A and chain B on the 170–180 stretch. This element is part of the apical α‐helix 6, a structurally flexible component already proposed to be PLP‐sensitive in AADC [30] and homologous a‐decarboxylases [34, 35].

As for WT, R347Q, and L353P, active sites were asymmetrically modeled with one open CL (chain A, in which Tyr332 is distant from PLP) and one closed CL (chain B) with the catalytic Tyr332 in proximity to the active site cleft.

In detail, the R347Q loop3 profile of fluctuations shows that the CL‐out (in chain A) behaves similarly to the WT, being in a rigid conformation, except for the second replica in which CL residues 331–340 show higher flexibility (Fig. 6C). Despite this, all replicas show a CL‐out conformation similar to the WT, and the interactions of substituted Gln347 with Asp345 are maintained (Fig. 6C, Fig. S4C,E). The CL‐in conformation shows higher flexibility than the CL‐out (Fig. 6C). Even if the global conformation of loop3 is unaltered in the three replicas, the catalytic Tyr332 is more distant from the PLP (Fig. S4A) with respect to the WT, and the RMSF values indicate that a stretch of loop3 (329–334) becomes more rigid (Fig. 6C).

The RMSF profile of the L353P variant, which maps at the end of loop3, shows that the Leu‐to‐Pro substitution does not significantly impact the local conformation of loop3. The RMSF profile for all chains is similar to that of R347Q (Fig. 6B). As for the latter, the CL‐out conformation is rigid and shows no main differences with respect to the WT (Fig. 6D). The RMSF values of CL‐in loop3 conformation suggest that this region is more flexible than the CL‐out conformation and similar to that of the WT (Fig. 6D, Fig. S4D,F). As for R347Q, the distance between Tyr332 and PLP (OH‐C4′) in L353P is larger than in the WT (Fig. S4B).

Overall, all‐atom MD simulations show that both catalytic variants exhibit a defect in loop3 mobility that leads to the catalytic Tyr332 mispositioning from PLP. This altered mobility could be the molecular basis for the catalytic inability. Since crystal structures and MD simulations as well as dynamic light scattering analyses (Table S2) do not show evident differences in the global molecular sizes of the species (WT [30, 36] and variants), we carried out an extensive SAXS analysis to reveal modifications of shape in solution.

### SAXS analyses show that R347Q and L353P variants affect some geometrical parameters of the dimeric species

**Fig. 7:**
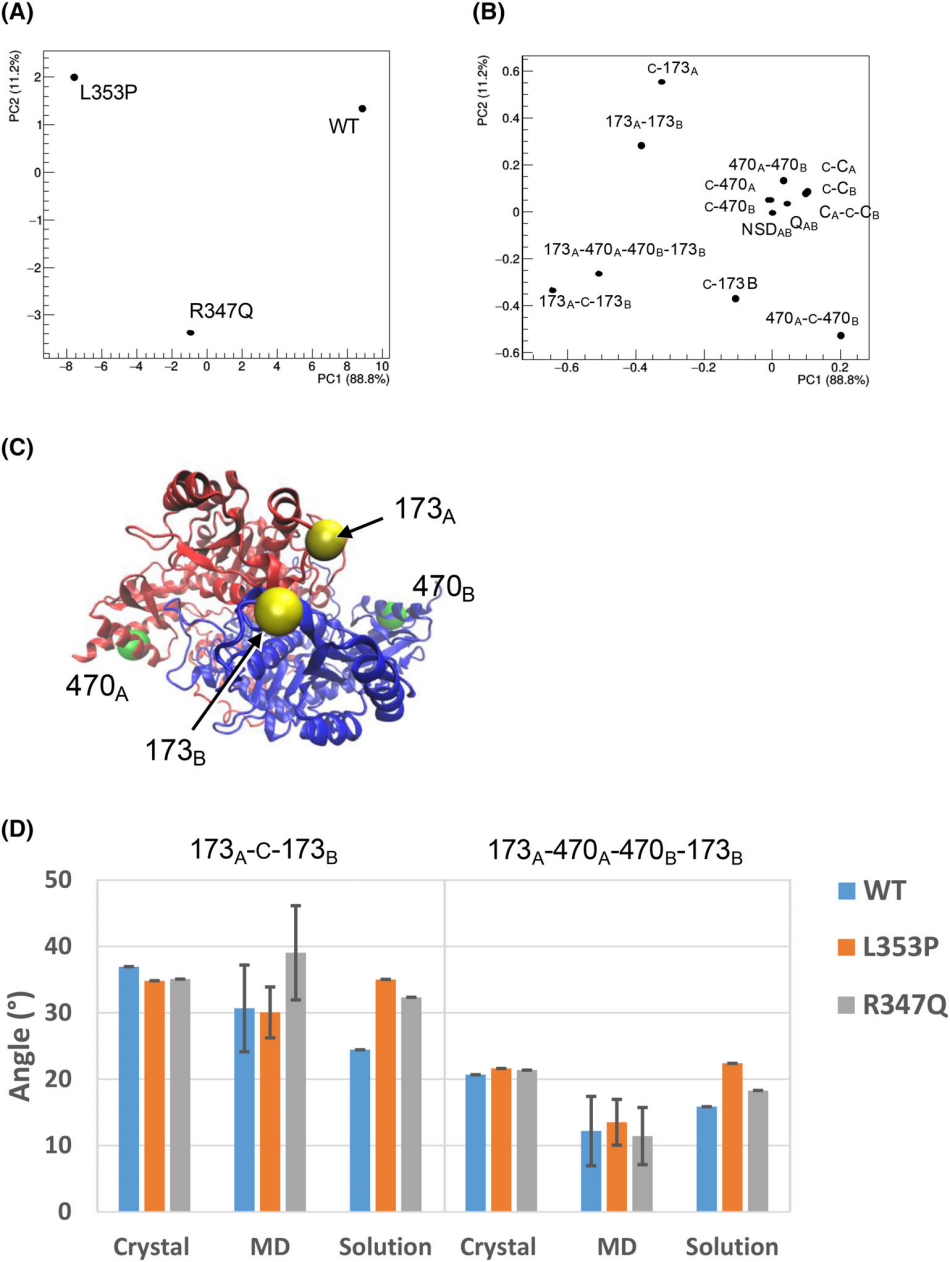
Comparison among WT, R347Q, and L353P atomistic models in best agreement with solution scattering data. (A) Scores and (B) loadings of the first two principal components (PC1 and PC2) calculated by principal component analysis applied to geometrical properties calculated for each atomistic model, showing that most of the geometrical variables are grouped around the origin of the PC2 vs PC1 plane, indicating a negligible contribution to the separation, as shown by the two variables describing the asymmetry between chain A and chain B (NSD_AB_ and Q_AB_). (C) AADC dimer (obtained from MD) with chain A and chain B colored respectively in red and blue, and residues 173 (yellow) and 470 (green) shown as beads. The figure has been generated by using the program vmd [62]. (D) Bar graph of the values of the angle 173_A_‐C‐173_B_ formed among residue 173 of chain A (173_A_) and chain B (173_B_) and the center of mass of the AADC dimer (C) and of the dihedral angle 173_A_‐470_A_‐470_B_‐173_B_ calculated for the experimental crystal and solution structures, and averaged considering all the frames of the molecular dynamics trajectories. Error bars indicate the standard deviation of the MD values.

The structural characterization of WT and R347Q and L353P variants by SAXS does not reveal substantial differences in the global shape of the AADC dimer (Table S3), indicating that its overall shape is conserved in solution. However, an in‐depth analysis of the structural features, carried out by combining experimental data with computational predictions, revealed an impaired interaction between chain A and chain B of the AADC dimer in both variants with respect to the WT. The frames of the MD trajectories are checked against SAXS data (Figs S5 and S6) and the atomistic model in best agreement with SAXS data is identified (Fig. S7). Thereafter, the main geometrical features of the SAXS‐validated models from WT and variants are compared by principal component analysis (PCA) (Fig. 7A,B). Results show that chain A and chain B of the AADC dimer in solution are more separated in R347Q and L353P variants than in WT. This occurs as a result of a rotational motion around an axis passing through the residues Leu470 of both chains, which increases the distance between residues Glu173 of both subunits in the dimer (Fig. 7C). Interestingly, Glu173 belongs to α‐helix 6, which has already been shown in the MD simulations to be less mobile with respect to the WT and sensitive to modifications in the PLP binding site [30]. It can be noted (Fig. 7D) that this feature is not present in crystal structures (WT PDB ID: 8OR9; R347Q PDB ID: 9HRH; L353P PDB ID: 9HRI) which is probably due to the strong intermolecular contacts within the crystal lattice (see Fig. S8).

In order to determine if local fluctuations in loop3 are associated with global dimer movements, we first correlated motion among residues within MD trajectories in WT AADC. This analysis reveals that local fluctuations in the most exposed regions of loop3 are highly anti‐correlated to movements of residues located in α‐helix 6 of the same chain comprising residue Glu173 (Fig. S9). Thus, L353P and R347Q variants can locally alter the correlated motion, slightly quenching it (Fig. S9A–C), probably due to the loosening of interchain interactions caused by the separation of the two chains due to the hinge motion around the 470A‐470B axis (Fig. S9D).

## Discussion

The need for a model of AADC deficiency in a cell line of neuronal origin prompted us to develop a KO for the *DDC* gene in the neuroblastoma SH‐SY5Y cell line. In this study, we obtained a *DDC*‐KO model that recapitulates some key features of AADC deficiency in terms of enzyme activity loss and modifications of the l‐dopa/dopamine metabolic profile. In addition, the *DDC*‐KO cells allowed us to model the behavior of two catalytic variants (R347Q and L353P). Together with a combinatory structural approach, we defined the molecular basis for their loss‐of‐function.

The reasons underlying the choice of the SH‐SY5Y cell line, widely used as a model for Parkinson's disease and other neurological disorders, are due to the fact that these cells exhibit numerous biochemical and functional characteristics of neurons, such as the expression of neurofilament proteins, dopamine transporter and receptors, and enzymes involved in the biosynthesis and clearance of dopamine [37, 38, 39]. Notably, we found that abolishing AADC expression in SH‐SY5Y cells does not affect the proliferation but makes them more sensitive to the cytotoxic effect of l‐Dopa. The higher sensitivity of *DDC‐KO* cells to l‐dopa could be explained by its accumulation and consequent autoxidation accompanied by an increase in oxidative stress, as demonstrated in other studies [40, 41].

Previous studies suggest that AADC dysfunction could impact the neuronal levels of key enzymes involved in dopamine metabolism [21]. Our work provides evidence that depletion of AADC increases the expression of COMT and MAOB, while the expression of MAOA and DBH is downregulated. The decrease in MAOA protein expression has also been reported in a *DDC* knock‐in mouse model [18], which could contribute to lower dopamine degradation. Additionally, it is known that the inhibition of MAO activity also reduces the AADC activity in rat brains [42], implying regulatory mechanisms between these enzymes. In contrast, TH expression is barely detected in both control and *DDC*‐KO cells. The low TH expression in the SH‐SY5Y cell line has been reported in numerous studies at mRNA [37] and protein levels even after the cells were differentiated with retinoic acid [28]. Indeed, the inability of SH‐SY5Y cells to synthesize or store dopamine may be attributed to this low expression of TH [28]. This assumption is consistent with another study where the overexpression of TH in SH‐SY5Y cells was enough to restore their basal dopamine production [43]. Possibly, the modulation in the expression of these key enzymes represents an adaptive mechanism of neuroblastoma cells to compensate for the synthesis and clearance of dopamine metabolites; however, further investigation is required to elucidate if these differences in protein expression are reflected in the enzymatic activity.

Since AADC deficiency is associated with alterations in the CSF and plasma monoamine profiles (low levels of HVA and DOPAC, accompanied by an increase in 3‐OMD) [21], we found that l‐dopa supplementation in *DDC*‐KO cells recapitulated the key alterations of monoamine metabolism of AADC deficiency, with a significant increase of 3‐OMD and absent production of dopamine downstream metabolites [2, 3]. This phenotype was rescued with the overexpression of the WT AADC protein, increasing dopamine biosynthesis and reducing 3‐OMD levels. Despite the increase of AADC activity and protein expression in cell lysates, the WT‐transfected *DDC‐KO* cells did not produce higher levels of dopamine, 3‐MT, DOPAC, or HVA than control cells. These lower levels of metabolites could be explained by the differences in the expression of the other enzymes involved in dopamine metabolism between control and *DDC‐KO* cells, as well as the missing or altered feedback mechanisms in the production and clearance of these monoamines.

We then examined the effects of transfection with cDNA of the catalytic variants R347Q and L353P. In both cases, activity is deeply lost and the amount of detectable dopamine is insignificant and in agreement with the values of catalytic efficiencies measured for the respective purified recombinant species [24, 25, 26]. The R347Q and L353P transfected *DDC*‐KO show that AADC loss‐of‐function reflects in alterations at the levels of metabolites of the l‐dopa/dopamine pathway.

The added value of this cell model with respect to the purified recombinant species is that it could reflect the features of the metabolic phenotypes, evaluating the levels of biomarkers. It could also provide a basis to evaluate the efficacy of possible precision therapeutic choices. Indeed, the new *DDC*‐KO cell model is effective in highlighting the alterations induced by pathogenic variants known to cause a severe phenotype, even if it is limited in determining the molecular cause for the altered metabolic phenotype. We thus associated the structural data obtained from the purified species with the results obtained in the cell model.

We report here the first crystal structures of AADC deficiency catalytic variants (R347Q and L353P) located in loop3 [24, 26, 30] which do not show rough differences to WT. This is also supported by the similarity of the spectroscopic signals collected with the recombinant species. The main modification is confined to an increase in the flexibility of the structural element loop3, an unstructured segment that organizes in WT upon substrate binding by correctly positioning the catalytic Tyr332 [30] and, as a consequence, allows the chemical reaction of dopamine synthesis to occur. All‐atom MD simulations and SAXS analyses reinforce that the impairment lies in loop3 for both variants. Since its mobility is altered, a mispositioning of the catalytic Tyr332 takes place. In addition, a reduced flexibility of some structural elements, such as α‐helix 6, associated with the active site by long‐range interactions [30] is also evident. Thus, it can be argued that amino acid substitutions at loop3 alter the mobility of the catalytic loop as well as the flexibility of regions far from but connected to the active site. SAXS data allow us to uniquely select specific snapshots for each MD trajectory, which exhibit a specific signature, i.e., a larger separation of the AADC chains in variants with respect to the WT. The hinge motion between the two chains is witnessed by large variations of the 173_A_‐C‐173_B_ and 173_A_‐470_A_‐470_B_‐173_B_ variables, being Glu173 (belonging to α‐helix 6) being a sensitive “probe” of events occurring at the active site. We interpret that the higher flexibility of loop3 could act as a lever for the modifications at the dimer interface (α‐helices 6 separation among the two subunits) with structural consequences at the active sites. Indeed, spectroscopic analyses show that the external aldimine with the l‐dopa analogue DME is incorrectly placed.

This determination paves the way for the possible screening of small molecules, which foster dimer closure and trigger productive structural dynamics. Since the two active sites are in communication through a polar cavity, as recently reported [30], we provide here the structural constraints for a bioinformatic search of possible compounds. As a consequence, promising small molecules could be screened for possible therapeutic development.

Overall, the *DDC*‐KO cell model provides a new system to readily study the effects of known and newly identified pathogenic variants in an intracellular neuronal environment, and it could be an appropriate model to determine the effect of potential therapeutic agents screened on the basis of the molecular alterations induced by the protein variants. Nonetheless, it could also be useful for other diseases, like Parkinson's disease, where dopamine is depleted.

## Materials and methods

### Materials

PLP, l‐Dopa, dopamine, PLP hydrate, l‐Dopa, DOPAC, HVA, 3‐methoxy‐l‐tyrosine monohydrate, 3‐MT hydrochloride, dopamine hydrochloride, 4‐hydroxy‐3‐methoxyphenyl‐D3‐acetic‐D2‐acid solution, dopamine‐1,1,2,2‐D4 hydrochloride, isopropyl‐β‐D thiogalactopyranoside (IPTG), phenylmethylsulphonyl fluoride (PMSF), trichloroacetic acid (TCA), PEG200, HEPES‐HCl were purchased from Sigma‐Aldrich (St. Louis, MO, USA). All other chemicals are of the highest purity available.

### Cell culture

SH‐SY5Y neuroblastoma cells (ATCC CRL‐2266; RRID: CVCL_0019) were cultured in high‐glucose Dulbecco's modified Eagle's medium (DMEM) containing sodium pyruvate (0.11 g·L^−1^), d‐glucose (4.5 g·L^−1^), and GlutaMAX supplemented with 10% fetal bovine serum (FBS), 100 U·mL^−1^ penicillin, and 100 μg·mL^−1^ streptomycin (Life Technologies, Gibco, Carlsbad, CA, USA). Cells were incubated at 37 °C with 5% CO_2_. All experiments were performed with Mycoplasma‐free cells.

### Generation of single cell‐derived *DDC* knockout clones with CRISPR/Cas9

CRISPR‐Cas9 kit targeting the second exon of the *DDC* gene was obtained from Origene (Rockville, MD, USA; cat. no. KN401345) and the single cell‐derived knockout clones were generated following the manufacturer's instructions with minor modifications. In this system, two gRNAs target exon 2 in the *DDC* locus, and the cutting site is repaired by the integration of a donor template DNA containing a puromycin resistance gene as a selection marker. Briefly, SH‐SY5Y cells were plated in 3.5 cm dishes and the next day subjected to co‐transfections of each gRNA (1 μg) together with the donor cassette (1 μg) using Lipofectamine™ 2000 reagent (Invitrogen, Waltham, MA, USA) following the manufacturer's instructions. As experimental controls, we added untransfected cells and cells transfected with pEGFPN1 vector to assess transfection efficiency. After 48 h, transfected SH‐SY5Y cells were diluted in 15 cm Petri dishes and incubated with 0.8 μg·mL^−1^ of puromycin to allow colony selection for the cells that had integrated the donor cassette. Medium was changed every 3 days containing fresh puromycin, and after 3 weeks, single colonies were picked. Transfections were carried out in duplicates, allowing us to collect also pools of colonies from which we extracted genomic DNA and total RNA using Trizol reagent (Invitrogen). Preliminary screening of pools derived from both gRNAs by PCR revealed the presence of the integrated donor cassette in both orientations. The puromycin‐resistant cells were isolated into single cells by limiting dilution method in a 96‐well plate and cultured in a media supplemented with 20% FBS. Once the well was confluent, cells were split into a 48‐well plate. Clones were grown until the number of cells was enough for liquid nitrogen storage and validation by western blot. *DDC* knockout clones showing the complete absence of AADC protein were further characterized by PCR and the analysis of *DDC* sequence in the sgRNAs target region using Sanger sequencing. *DDC* exon 2 was amplified with PfuUltra High‐Fidelity DNA polymerase (Agilent Technologies, Santa Clara, CA, USA) using the following primers: 5′‐AGGTGTCCCTACCCACGGCT‐3′ (forward) and 5′‐GCCCTCCTGTTTTCTGACCTTGGA‐3′ (reverse). PCR conditions were: 95 °C for 2 min; 35 cycles consisting of 95 °C for 40 s, 58 °C for 40 s, 72 °C for 3 min; 72 °C for 10 min. The PCR products were separated on 2% agarose gel and then purified using the GenElute™ Gel Extraction kit (Sigma‐Aldrich). Purified DNA was cloned into the TOPO vector and transformed into *Escherichia coli* (Zero Blunt TOPO® PCR cloning kit for sequencing; Life Technologies). Next, single colonies were picked, and the plasmids were reisolated by miniprep (QIAprep® Spin kit; Qiagen, Hilden, Germany) and then submitted for sequencing (Eurofins Genomics, Ebersberg, Germany).

### Real‐time PCR (qPCR)

Total RNA was isolated from 1 × 10^6^ cells using TRIzol Reagent (Life Technologies). The cDNA synthesis was performed with 1 μg of RNA using the high‐capacity cDNA reverse transcription kit (Applied Biosystems, Waltham, MA, USA), following the manufacturer's instructions. qPCR was performed using GoTaq® qPCR Master Mix (Promega, Madison, WI, USA) on a QuantStudio 3 Real‐Time PCR system (Thermo Fisher Scientific, Waltham, MA, USA). The relative expression mRNA levels were calculated with the $2^{-\Delta \Delta C_{t}}$ method using glyceraldehyde‐3‐phosphate dehydrogenase (GAPDH) as an endogenous control. The following pairs of primers were used: *DDC*, 5′‐ACCACAACATGCTGCTCCTTTG‐3′ (forward) and 5′‐CATTCAGAAGGTGCCGGAACTC‐3′ (reverse). *GAPDH*, 5′‐ATCAGCAATGCCTCCTGCAC‐3′ (forward) and 5′‐TGGTCATGAGTCCTTCCACG‐3′ (reverse).

### Cell proliferation and viability assay

For cell proliferation, cells were seeded in 24‐well plates at a density of 2 × 10^4^ cells per well and grown at 37 °C with 5% CO_2_. Next, cells were counted every 2 days using the trypan blue exclusion method. Cell viability was determined using the OZBlue kit (OZ Biosciences, San Diego, CA, USA). Cells were plated in a 96‐well plate at a density of 2 × 10^4^ cells per well and treated with different concentrations of l‐dopa or dopamine for 24 h. OZBlue reagent was added directly into the culture medium and cells were incubated for 1 h under standard conditions. After incubation, the fluorescence (560 nm_Ex_/590 nm_Em_) was measured using a multimode microplate reader (GENios; Tecan, Männedorf, Switzerland).

### Protein extraction and western blotting

To prepare the samples, cells were collected in ice‐cold 1× PBS (Life Technologies, Gibco) and centrifuged at 600 ***g*** for 5 min. Pellets were resuspended in RIPA lysis buffer system® (Santa Cruz, Dallas, TX, USA) according to the manufacturer's protocol. The lysate was centrifuged at 5000 ***g*** for 10 min at 4 °C. The protein concentration in the supernatant was determined using Bradford reagent (PanReac AppliChem, Darmstadt, Germany). After boiling for 5 min in 4× Laemmli sample buffer, protein samples (20 μg) were separated on 12% polyacrylamide gels and electrotransferred to PVDF membrane (Merck Millipore, St. Louis, MO, USA). Ponceau S (Sigma‐Aldrich) was used to assess protein transfer. Membranes were blocked with 5% milk in 1× Tris‐buffer saline pH 7.4 (50 mm Tris, 150 mm NaCl) with 0.1% Tween (1× TBST) for 1 h at room temperature. Then the membranes were incubated with a primary antibody overnight at 4 °C: DDC (SC‐293287, 1 : 2000; Santa Cruz); Vinculin (SC‐25336, 1 : 2000; Santa Cruz); COMT (A4435, 1 : 1000; ABclonal, Dusseldorf, Germany); DBH (A23579, 1 : 1000; ABclonal); MAOA (A4105, 1 : 1000; ABclonal); MAOB (A11597, 1 : 1000; ABclonal); TH (A5079, 1 : 1000; ABclonal); TH (SC‐25269, 1 : 1000; Santa Cruz). The next day, membranes were washed three times with 1× TBST and incubated in secondary goat anti‐rabbit HRP antibody (AS014, 1 : 5000; ABclonal) and anti‐mouse IgG Fc BP‐HRP antibody (SC‐525409, 1 : 5000; Santa Cruz) for 1 h at room temperature and washed three times with 1× TBST. HRP signal was developed using Wester Antares chemiluminescence substrate (Cyanagen, Santa Clara, CA, USA). Images were taken on ChemiDoc imaging system (Bio‐Rad, Hercules, CA, USA). Densitometric analysis was done using imagej (National Institute of Health, Bethesda, MD, USA).

### AADC activity assay

The enzymatic activity of AADC was measured as previously described [21] with minor modifications. Cells (2 × 10^6^) were washed twice with 1× PBS, harvested, and centrifuged at 600 ***g***. Pellets were resuspended with lysis buffer pH 7.4 containing 10 mm Tris (Carl Roth, Karlsruhe, Germany), 1 mm EDTA (Sigma‐Aldrich), 320 mm sucrose (Sigma‐Aldrich), and 1× protease inhibitor mix M (SERVA Electrophoresis, Heidelberg, Germany). Samples were snap‐freezed twice in liquid nitrogen and protein concentration was determined using the Bradford reagent. Cell lysates were incubated with assay buffer pH 7.4 (70 μm pyridoxal phosphate, 0.1 m potassium phosphate, and 0.167 mm EDTA) for 1 h at 37 °C. Subsequently, 2 mm final concentration of l‐dopa (Sigma‐Aldrich) was added and incubated for 15 min at 37 °C. The reaction was stopped by adding trichloroacetic acid (final concentration 0.6 mm). Samples were centrifuged at 5200 ***g*** for 10 min at 4 °C, and dopamine in the supernatant was quantified by high‐performance liquid chromatography. A sample blank without cell lysate, a substrate blank without l‐dopa, and a reaction blank with trichloroacetic acid from the start were performed for each sample. Activity was measured as previously reported [36].

### Site‐directed mutagenesis and purification in bacterial cells and site‐directed mutagenesis for transient transfection in *DDC*‐KO cells

R347Q and L353P variants were cloned, expressed, and purified as already described [24, 26]. Constructs of human AADC pathogenic variants were generated based on the pCMV‐*DDC* expression vector (Origene Technologies). The mutations were inserted using the QuickChange II site‐directed mutagenesis kit (Agilent) with the following oligonucleotides: for the L353P variant, 5′‐CATTGGCAGATACCACCGGGCAGAAGATTT‐3′ and its reverse complement; for the R347Q variant, 5′‐CTTATCACTGACTACCAGCATTGGCAGATAC‐3′ and its reverse complement. All mutations (the mutated codons are underlined) were confirmed by DNA sequencing. For plasmid transfections, the *DDC*‐KO cells were transfected using Lipofectamine 3000 (Invitrogen) with 2 μg of DNA and incubated for 2 days, following the manufacturer's instructions.

### Mass spectrometry for quantification of dopamine and its metabolites

For the quantification of dopamine and downstream metabolites, the WT and *DDC*‐KO cells were plated in 100 mm Petri dishes. Since dopamine metabolites were not detectable in WT or *DDC*‐KO clones in basal conditions independently of the number of cells, the following day, cells were washed twice with 1× PBS and treated for 2 h with 80 μm l‐dopa in phenol‐red free medium supplemented with 1% FBS. For extracellular metabolites, a sample of culture medium was diluted 1 : 10 in ice‐cold 90% methanol. Samples were centrifuged at 18 000 ***g*** and 4 °C for 10 min, and the supernatant was recovered for LC–MS analysis. For intracellular metabolites, cells were rinsed once with ice‐cold 1× PBS, collected by cell scraper in 500 μL of ice‐cold 80% methanol, and transferred into prechilled microcentrifuge tubes. After centrifugation at 18 000 ***g*** and 4 °C for 10 min, the supernatant was recovered for LC–MS analysis. The pellets were resuspended in 100 μL of denaturing solution (8 m urea, 100 mm ammonium bicarbonate) and protein concentration was determined using Bradford Reagent. LC–MS/MS analysis was performed by means of an Agilent 1290 Infinity UHPLC® (Ultra High‐Performance Liquid Chromatography) coupled with a quadrupole mass detector 6495 (Agilent) equipped with an electrospray ion source (Jet Stream ESI). Chromatographic separation was achieved using a ZORBAX Eclipse plus C18 column (3.0 mm width, 50 mm length, 1.8 μm particle size) (Agilent) with the column temperature of 40 °C. Nitrogen was employed as an ion source gas and collision gas. For dopamine, 3‐OMD and 3‐MT the mobile phase consisted of A: Water + 0.1% TFA + 10 mm NH_4_F; B: methanol, with the following gradient (flow, 0.50 mL·min^−1^): 0.00–4.00 min, 35%B; 4.01–5.00 0%B; post‐time 1.0 min. The optimized parameters were: capillary voltage 1.5 kV; V charging 0 V, Delta EMV 600 V, gas temperature 250 °C; sheath gas 300 °C; drying gas 11 L·min^−1^; sheath flow 12 L·min^−1^. Multiple reaction monitoring in positive mode was employed. Mass transitions were (*m/z*) 154 > 137 (collision energy, CE 8 V) and 154 > 91 (CE 28 V) for dopamine; *m/z* 158 > 141 (CE 8 V) and 158 > 95 (CE 28 V) for dopamine‐d3; 212 > 166 (CE 19 V) and 212 > 149 (CE 10 V) for 3‐OMD; *m/z* 151 > 119 (CE 9 V) and 151 > 91 (CE 17 V) for 3‐MT. The chromatographic separation for HVA and DOPAC was performed by the same column. The mobile phase consisted of A: Water + 0.2% FA; B: methanol, with the following gradient (flow, 0.500 mL·min^−1^): 0.00–0.5 min 10% B; 0.51–5 = 3.00 60% B; 3.01–4.00 95% B; 4.01–5.00 10% B; post‐time 1.0 min. The optimized parameters were: capillary voltage 3.5 kV; V charging 1.5 kV, Delta EMV 600 V, gas temperature 290 °C; sheath gas 300 °C; drying gas 1 L·min^−1^; sheath flow 11 L·min^−1^. Multiple reaction monitoring in negative mode was employed. Mass transitions were (*m/z*) 167 > 123 (CE 3 V) for DOPAC; *m/z* 186 > 142 for HVA‐d4 (CE 3 V); *m/z* 181 > 122 (CE 10 V) for HVA.

All data were acquired and processed by masshunter workstation Software B.07.01 version (Agilent).

### Crystallization of R347Q and L353P variants, data collection and processing

Purified R347Q and L353P variants were prepared at a concentration of about 20 mg·mL^−1^ in 50 mm HEPES pH 7.4. The crystallization conditions were similar to those previously reported for human holoAADC [30] using the hanging‐drop vapor diffusion method at 20 °C. The best crystals were obtained in 0.1 m HEPES pH 8.0 and 40% PEG 200 by mixing 1.5 μL of protein solution with 0.5 μL of reservoir; diffraction‐quality crystals were obtained in about 7 days.

Data for both the R347Q and L353P variants were collected at the XRD2 beamline of the Elettra synchrotron in Trieste, Italy (λ 1.0 Å), at 100 K after a brief soaking in a mixture of 70% mother liquor and 30% glycerol. Data were indexed, integrated, reduced, and converted to structure factors using mosflm [44], scala [45], and TRUNCATE from the ccp4 suite [46].

### Structure determination and refinement

Both the R347Q and L353P variants were modeled into difference Fourier maps phased by the structure of the WT native AADC (PDB ID 8OR9). Both models were rigid body refined, initially moving the entire molecule and, in a second stage, the elements of secondary structure using the program phenix.refine [47, 48], as implemented in the phenix suite [49]. After the proper side chains for the mutated amino acids had been introduced, the models were subjected to a series of rounds of positional refinement alternated with manual model revisions with coot [50] and phenix.refine. The models were finally subjected to final rounds of TLS refinement [51] and solvent molecules were added to both the models of the R347Q and L353P variants in the final stages according to hydrogen bond criteria and only if their B‐factors refined to reasonable values and if they improved the R‐free.

During the process of refinement and model building, the quality of the models was controlled with molprobity [52]. The diffraction data and refinement statistics of the models are summarized in Table S1.

### Modeling of holo form of L353P and R347Q AADC variants for MD simulations

The R347Q and L353P structural models were generated through *in silico* mutation of the holo AADC‐WT model, previously reported [30]. The Rotamers tool [53] within the ucsf chimera [54] program was employed in this mutation process. The topology parameters and atomic partial charges were assigned following the methodology reported [30].

### All‐atom MD simulations

All‐atom MD simulations of R347Q and L353P variants were carried out utilizing the gromacs program, version 2019 [55]. These simulation parameters were consistent with those employed in the previous study to ensure comparability of results [30]. The systems were solvated with water using the TIP3P water model [56, 57] and ion molecules (0.154 m Na^+^/Cl^−^ to mimic physiological conditions) were added. Each system underwent a comprehensive equilibration process, including 5000 steps of steepest descents minimization, followed by NVT equilibration for 100 ps, NPT equilibration for another 100 ps, and MD production under the NPT ensemble for 1000 ns. Simulations were executed with a time‐step of 2 fs, maintaining a coupled temperature of 300 K using the V‐rescale thermostat [57] and a coupled pressure of 1 bar using the Parrinello–Rahman barostat [58]. For each species, three replicas of 1 μs were performed and data after 500 ns of equilibration were analyzed and compared to the WT AADC [30]. A structure representative for each MD trajectory was obtained using the “cluster” tool from the gromacs package, applying the gromos algorithm with a cut‐off of 30 Å [59].

### Study of correlated motion between loop 3 and α‐helix 6

The relationship between local fluctuations in loop3 and global movements of the dimer chains has been investigated by analyzing the correlated motion of C_α_ atoms along the MD trajectories through the dynamics cross‐correlation matrix (DCCM).

### SAXS measurements and data analysis

SAXS measurements were performed during two beamline sessions: the first at ESRF, beamline BM29, carried out in batch mode, the second at Diamond Light Source, beamline B21, carried out by using in‐line size‐exclusion chromatography (SEC‐SAXS). Batch mode measurements were performed on samples of the holo forms of WT, R347Q, and L353P variants. Samples at protein concentration ranging from 1 to 10 mg·mL^−1^ were picked up from batch plates stored at 4 °C by using the automated sample changer EMBL Arinax sample handling robot. To reduce radiation damage, samples were analyzed under continuous flow during SAXS measurements. For each sample, 25 frames were acquired for each concentration with an acquisition time of 1 s per frame (total exposure time of 25 s). Buffer measurements before and after each sample were acquired for background subtraction. SEC‐SAXS measurements were performed on WT and L353P samples, buffer exchanged against 0.5 m NaCl, 20 mm Tris–HCl (pH 8.5), and 5 mm DTT by using an Amicon‐4 Centrifugation Unit (cut‐off 10 kDa) and concentrated up to 4 mg·mL^−1^ just before data collection to avoid sample aggregation and/or degradation. SEC‐SAXS data collections were performed, processed, and analyzed as already described [30]. Experimental data and *ab initio* models were deposited in SASBDB (SASDW74 and SASDW84 for L353P and R347Q AADC forms, respectively).

### Modeling of SAXS data

The atomistic models generated by MD (see above) were compared with SAXS profiles by using the program crysol [60]. Each individual frame of the MD trajectories was checked against SAXS data, i.e., it was used for calculation with crysol to fit the 1D‐experimental curve with that calculated from the MD‐simulated AADC model. For the same protein sample, all the MD replicas were considered, and they were checked against batch and SEC‐SAXS data. The χ^2^ value obtained from the fit was used to evaluate the model in best agreement with data. The agreement of the best models with SAXS data was also evaluated in direct space, by assessing their similarity with the molecular envelopes determined *ab initio* from SAXS data. This calculation was performed by using the supcomb program [61], which uses the normalized spatial discrepancy as distance metrics. The models in best agreement with SAXS data obtained for all enzyme species were compared by considering their geometric properties, calculated by using vmd [62] scripts developed *ad hoc*.

The following variables were considered:

- distances between the center of mass of the AADC dimer and the center of mass of its individual chains: C‐C_A_, C‐C_B_;
- distances between relevant residues for the AADC shape, i.e., Glu173 and Leu470, from the center of the dimer and from the center of its individual chains: C‐173_A_, C‐173_B_, C‐470_A_, C‐470_B_;
- distances between the residues Glu173 and Leu470 of the two chains of the AADC dimer: 173A‐173B, 470A‐470B;
- the angle formed by the center of mass of the AADC dimer and those of its two chains: CA‐C‐CB;
- the angle formed by the residue Glu173 of the two chains and the center of mass of the AADC dimer: 173A‐C‐173B;
- the dihedral angle formed by the residues Glu173 and Leu470 of the two chains of the AADC dimer: 173A‐470A‐470B‐173B;
- the asymmetry between chain A and chain B, measured by the normalized spatial discrepancy (NSD_AB_) and by the alignment quality (Q_AB_), was calculated by the programs supcomb and superpose [63], respectively.

The geometrical features from different models were analyzed via principal component analysis (PCA) by using the program rootprof [64].

### Spectroscopic measurements

CD measurements were recorded with a Jasco J‐715 spectropolarimeter (Jasco Europe, Cremella [LC], Italy) equipped with a Grant LTC2 refrigerated circulator at 25 °C in the presence of 100 μm PLP at a scan speed of 50 nm·min^−1^, band width 1 nm, response 4 s, data pitch 1 nm, cell length 1 cm. Near UV–visible spectra were recorded in 100 mm potassium phosphate, pH 7.4 at a protein concentration of 0.5 mg·mL^−1^ while far UV spectra were recorded in 30 mm potassium phosphate, pH 7.4 at a protein concentration of 0.1 mg·mL^−1^. Secondary structure content was determined in the 250–185 nm range using the BeStSel algorithm [65]. Thermal denaturation was performed in 100 mm potassium phosphate, pH 7.4 by monitoring the dichroic signal of 0.2 mg·mL^−1^ protein at 222 nm, with a band width of 1 nm, response 4 s, data pitch 0.2 °C, cell length 0.1 cm and with a temperature gradient of 90 °C·h^−1^ between 25 °C and 90 °C. Absorbance spectra were recorded on a Jasco V‐750 spectrophotometer equipped with a CTU‐100 thermostat unit in 100 mm potassium phosphate, pH 7.4, at 25 °C with a 0.5 mg·mL^−1^ protein concentration in the absence or in the presence of a saturating concentration of DME. All spectra and thermal curves were repeated in triplicate and results are reported as mean ± SD.

### Dynamic light scattering analyses

Dynamic light scattering measurements were obtained on a Zetasizer Nano ZS instrument (Malvern, Malvern, UK), using disposable ZEN0112 polystyrene cuvettes. Settings used for particle size measurements are reported in Ref. [36]. Samples were prepared at 0.4 mg·mL^−1^ protein concentration in 100 mm potassium phosphate, pH 7.4, with an additional 100 μm PLP. All samples were filtered using a 0.02 μm Anotop 10 filter (Whatman). Each single value derives from at least 40 measurements, each one consisting of 12–18 repetitions. All experiments were repeated in triplicate, and results were reported as mean ± SEM.

### Statistical analysis

Data are presented as the mean ± standard error of the mean (SEM) of at least three independent experiments (biological replicates). Statistical differences were determined by one‐way analysis of variance (ANOVA) for multiple data sets, followed by Dunnett's test when comparisons were performed to a single control. Statistical significance was defined as *P* < 0.05. Data were analyzed using prism 8.0 software (GraphPad, Boston, MA, USA).

## Conflict of interest

MB has received grants for research and consultation by PTC Therapeutics; all other authors have nothing to disclose.

## Author contributions

CAC‐C, GB, MP, RPR, AG, BDB, RC, and PM‐JL planned and performed experiments and analyzed data. RF, GL, and RG performed experiments. CAC‐C and GB wrote the preliminary draft. MB planned the work, analyzed data and interpreted results, searched for funding, and wrote the final form of the paper.

## Peer review

The peer review history for this article is available at https://www.webofscience.com/api/gateway/wos/peer‐review/10.1111/febs.70120.

## Supporting information


**Fig. S1.** Validation of *DDC* gene knockout.
**Fig. S2.** Spectroscopic and coenzyme microenvironment features of R347Q and L353P AADC variants.
**Fig. S3.** RMSD and gyration radius (Rg) determination by MD simulations of R347Q and L353P.
**Fig. S4.** Tyr332‐PLP distance, CL‐in and CL‐out conformations from MD simulations of R327Q and L353P AADC variants.
**Fig. S5.** SAXS profiles.
**Fig. S6.** *Ab initio* SAXS molecular envelopes superposed to the atomistic models in best agreement with SAXS data.
**Fig. S7.** χ^2^ values obtained by fitting each frame of the MD simulations against SAXS profiles.
**Fig. S8.** Values of angular variables related to the hinge motion of chain A and chain B of the AADC dimer calculated for each frame of the MD simulations.
**Fig. S9.** Dynamical cross‐correlation matrix (DCCM) of the AADC dimer calculated for each frame of the MD simulations for WT and L353P and R347Q variants.
**Table S1.** Crystallographic data collection and refinement statistics.
**Table S2.** Dimensional parameters of all AADC species determined dynamic light scattering measurements, crystal structure and MD simulations.
**Table S3.** Parameters estimated from SAXS data.

## Data Availability

The X‐ray diffraction data and crystal structures of human holo AADC R347Q and L353P variants can be found in the PDB databank with entry 9HRH and 9HRI, respectively. The SAXS experimental data and *ab initio* models can be found in the SASBDB databank with entry SASDW74 and SASDW84: experimental data and *ab initio* models for L353P and R347Q AADC forms, respectively.
