# Supplementary material for: The CRISPR‐Cas9 knockout DDC SH‐SY5Y in vitro model for AADC deficiency provides insight into the pathogenicity of R347Q and L353P variants: a cross‐sectional structural and functional analysis
Source: FEBS J. 2025 May 3;292(18):4833–53. doi: 10.1111/febs.70120 (PMC12443470; doi:10.1111/febs.70120)
Supplement: Supplementary file 1 — Fig. S1. Validation of DDC gene knockout. Fig. S2. Spectroscopic and coenzyme microenvironment features of R347Q and L353P AADC variants. Fig. S3. RMSD and gyration radius (Rg) determination by MD simulations of R347Q and L353P. Fig. S4. Tyr332‐PLP distance, CL‐in and CL‐out conformations from MD simulations of R327Q and L353P AADC variants. Fig. S5. SAXS profiles. Fig. S6. Ab initio SAXS molecular envelopes superposed to the atomistic models in best agreement with SAXS data. Fig. S7. χ2 values obtained by fitting each frame of the MD simulations against SAXS profiles. Fig. S8. Values of angular variables related to the hinge motion of chain A and chain B of the AADC dimer calculated for each frame of the MD simulations. Fig. S9. Dynamical cross‐correlation matrix (DCCM) of the AADC dimer calculated for each frame of the MD simulations for WT and L353P and R347Q variants. Table S1. Crystallographic data collection and refinement statistics. Table S2. Dimensional parameters of all AADC species determined dynamic light scattering measurements, crystal structure and MD simulations. Table S3. Parameters estimated from SAXS data. [file FEBS-292-4833-s001.pdf]

Supporting Information

Table S1. Crystallographic data collection and refinement statistics.

**AADC Data set**	**Holo human AADC Arg347Gln**	**Holo human AADC Leu353Pro**
Space group	$P6_122$	$P6_122$
Crystal form	1	2
a (Å)	107.27	107.25
b (Å)	107.27	107.25
c (Å)	218.85	219.19
α	90.0	90.0
β	90.0	90.0
γ	120.0	120.0
Molecules in the asymmetric unit	1	1
Resolution range (Å)	72.95 - 1.70	48.17 - 2.05
Observed reflections	673,179	455,055
Independent reflections	82,318	47,570
Multiplicity*	8.2 (8.1)	9.6 (9.7)
Rmerge (%)[a]	9.0 (47.2)	6.3 (53.0)
<I/s(I)>	11.6 (2.7)	24.6 (4.3)
Completeness (%)	100.0 (100.0)	100.0 (100.0)
Reflections in refinement	82,221	47,502
Rcryst (%)[b]	19.96	18.89
Rfree (%) (test set 5%)[c]	20.63	20.95
Protein atoms	3,655	3,529
Ligand atoms	41	41
Water molecules	353	234
R.m.s.d. on bond lengths (Å)[d]	0.003	0.003
R.m.s.d. on bond angles (Å)[d]	0.631	0.631
Planar groups (Å)[d]	0.005	0.005
Chiral volume dev. (Å^3)[d]	0.043	0.042
Average B factor (Å^2)	23.62	33.90
Protein atoms	22.90	33.59
Ligand atoms	31.91	44.02
Solvent atoms	30.04	36.84
Synchrotron beamline<br>Date of data collection	Elettra – XRD2<br>28/01/2020	Elettra – XRD2<br>22/07/2020

* The values in parentheses refer to the highest resolution shells.

[a] Rmerge = Σ*h*Σ*i* | I*ih* – <I*h*> / Σ*h*Σ*i* <I*h*> where <I*h*> is the mean intensity of the *i* observations of reflection *h*.

[b] Rcryst = Σ | |Fobs| - |Fcalc| | / Σ |Fobs|, where |Fobs| and |Fcalc| are the observed and calculated structure factor amplitudes, respectively. Summation includes all reflections used in the refinement.

[c] Rfree = Σ | |Fobs| - |Fcalc| | / Σ |Fobs|, evaluated for a randomly chosen subset of 5% of the diffraction data not included in the refinement.

[d] Root mean square deviation from ideal values.

Table S2. Dimensional parameters of all AADC species determined dynamic light scattering measurements, crystal structure and MD simulations. Data are reported as mean ± standard error of the mean (SEM) of at least three independent experiments.

**Species**	**Diameter (nm) by dynamic light scattering**	**Rg (nm) by X-ray structure (dimer)**	**Rg (nm) by MD simulations**
WT	9.61 ± 0.04[a,b]	2.78 [a]	2.85 ± 0.10 [a]
R347Q	9.77 ± 0.07	2.79	2.81 ± 0.01
L353P	9.60 ± 0.06	2.81	2.79 ± 0.01

[a]Bisello, G., Ribeiro, R. P., Perduca, M., Belviso, B. D., Polverino De' Laureto, P., Giorgetti, A., Caliandro, R. & Bertoldi, M. (2023) Human aromatic amino acid decarboxylase is an asymmetric and flexible enzyme: Implication in aromatic amino acid decarboxylase deficiency, *Protein Sci.* **32**, e4732.

[b]Bisello, G., Kusmierska, K., Verbeek, M. M., Sykut-Cegielska, J., Willemsen, M. A. A. P., Wevers, R. A., Szymańska, K., Poznanski, J., Drozak, J., Wertheim-Tysarowska, K., Rygiel, A. M. & Bertoldi, M. (2022) The novel P330L pathogenic variant of aromatic amino acid decarboxylase maps on the catalytic flexible loop underlying its crucial role, *Cell Mol Life Sci.* **79**, 305.

Table S3. Parameters estimated from SAXS data. Maximum momentum transfer (q_{max}), radius of gyration (R_g) from Guinier analysis (reciprocal space) and pair distribution function determination (real space), maximum inter-particle distance (D_{max}), Porod-Debye volume and molecular weight (MW) estimated by using the Porod approach are shown.

**Sample**	**Å^{-1}**	**R_g reciprocal space (Å)**	**R_g real space (Å)**	**D_{max} (Å)**	**Porod-Debye Volume (Å^3)**	**MW (kDa)**
WT	0.39	35	35	150	169490	145
WT SEC	0.26	33	33	120	160000	109
L353P	0.30	34	34	120	179090	143
L353P SEC	0.14	32	32	125	165910	111
R347Q	0.24	37	37	130	185950	151

SEC, size exclusion chromatography.

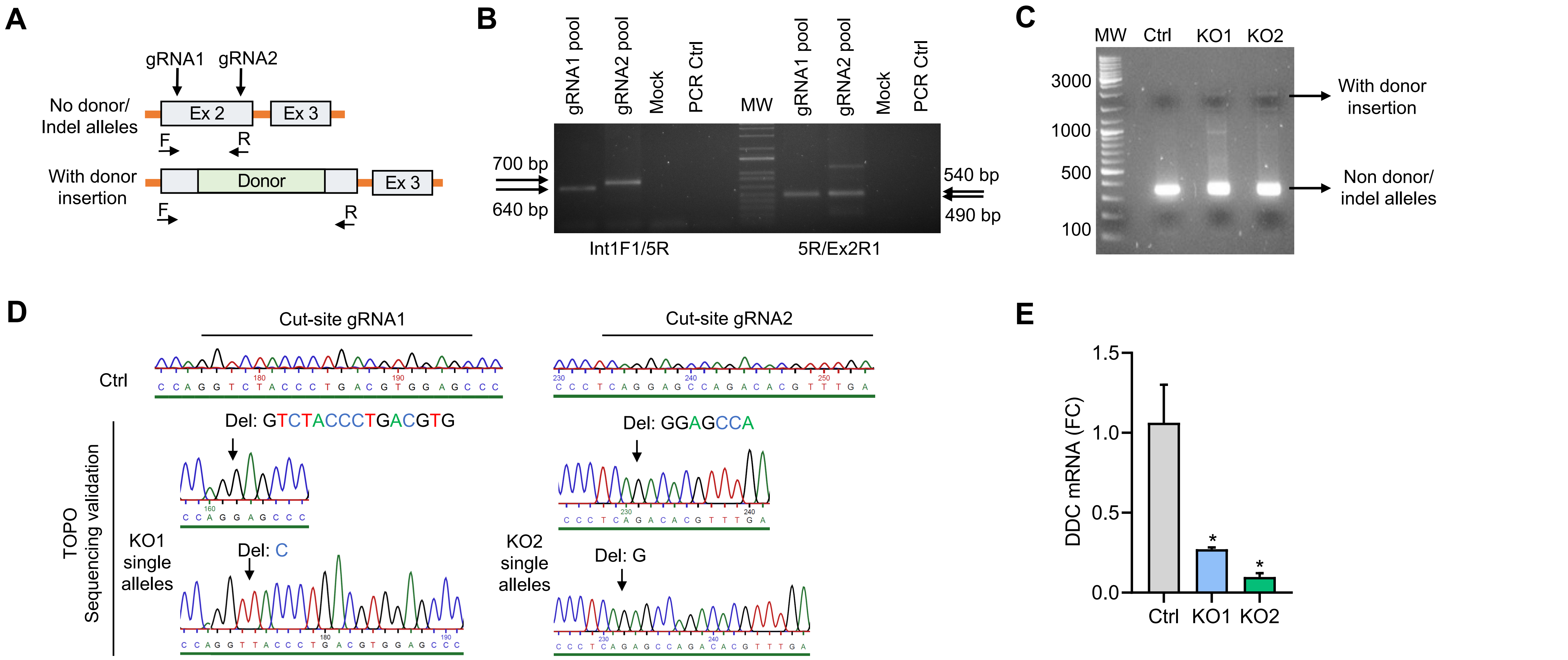

Figure S1. Validation of *DDC* gene knockout A) Scheme illustrating the exon 2 of *DDC* gene and sites targeted by guide RNAs. Half-arrows indicate the cut-site primers used to verify the presence of recombinant events. Forward (F) reverse (R). **B)** Verification of donor insertion in forward and reverse orientations in the pools of puromycin-resistant cells. PCR was performed using a primer annealing within the donor cassette (5R, sequence by Origene) coupled with either a primer located in the *DDC* intron preceding exon 2 (Int1F1: 5'-CCAGTGCCATTTCTACCTCCTAC-3') or a primer located in *DDC* exon 2 downstream to the insertion site (Ex2R1: 5'-CCAGGCATGATTATCTTCTCAAC-3'). **C)** Representative results from genomic PCR amplification to verify the presence of both donor-inserted and indel alleles. **D)** Sample results from Sanger sequencing of single alleles isolated by TOPO cloning. Both *DDC* knockout clones (KO1 and KO2) have heterozygous recombination events. **E)** Real-time PCR analysis of relative *DDC* mRNA expression levels. The values are reported as fold change (FC) relative to the control cells. Data are represented as mean ± SEM of three independent experiments. Asterisks indicate *$P < 0.05$ in comparison to the control cells.

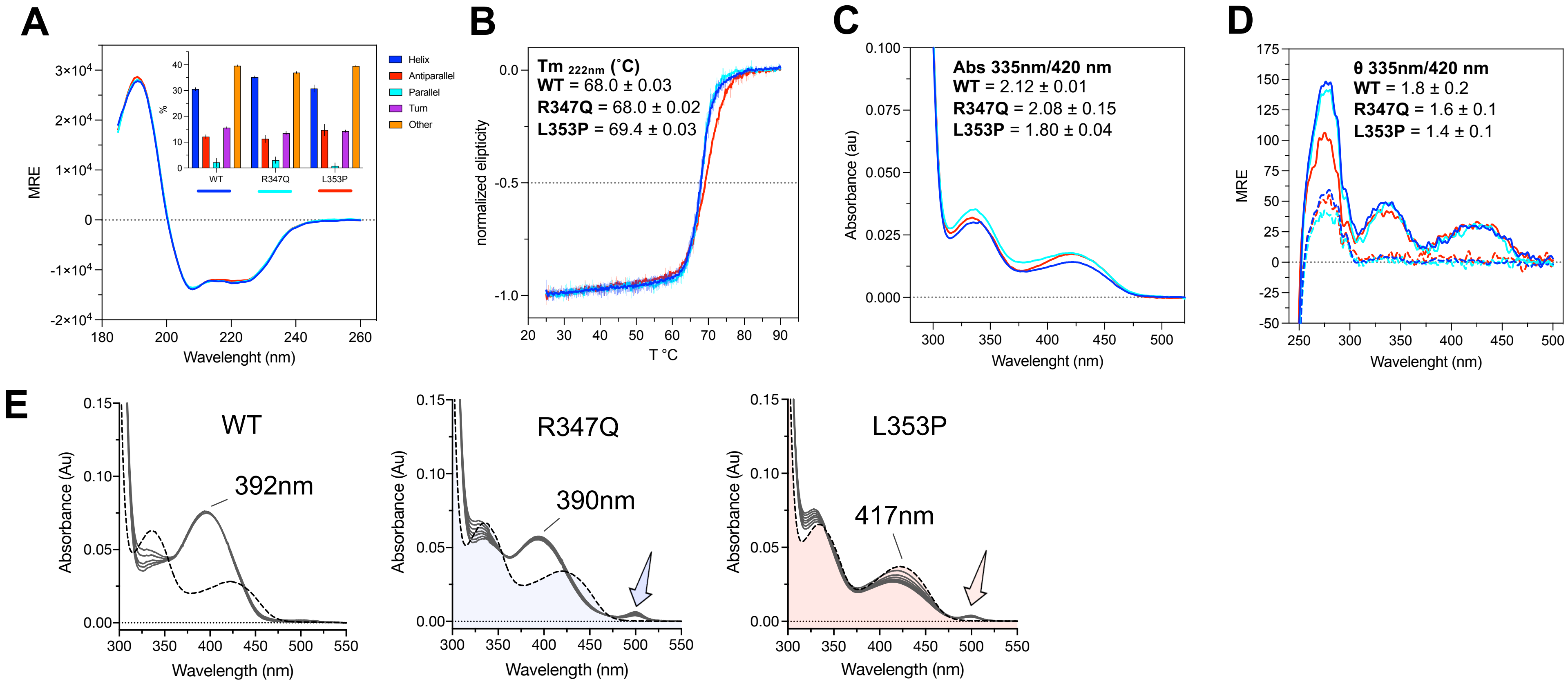

Figure S2: Spectroscopic and coenzyme microenvironment features of R347Q and L353P AADC variants. A) Far-UV CD spectra were recorded at 0.1 mg/mL protein concentration in 30 mM potassium phosphate buffer, pH 7.4, with the addition of 100 µM PLP. Each spectrum is the average of three spectra. In the inset, the results of the secondary structure content. The bars represent the mean ± SD result from the analysis of three spectra. Color codes: blue, WT; cyan, R347Q; red, L353P. **B)** thermal denaturation curves were obtained in 100 mM potassium phosphate buffer, pH 7.4 at 0.2 mg/mL protein concentration following the signal at θ 222 nm. Curves are normalized between two states, -1 (folded) and 0 (unfolded). Each curve is the average curve of three independent experiments. Calculated melting temperature are reported as mean±SD. **C)** Absorbance spectra were recorded 100 mM potassium phosphate buffer, pH 7.4, at 25 °C at 0.5 mg/mL protein concentration. Spectra are the average of three independent experiments and the tautomeric ratio ($A_{335nm}/A_{420\ nm}$) is reported as mean±SD. **D)** Near UV-visible CD spectra were recorded in 100 mM potassium phosphate buffer, pH 7.4, at 25 ◦C at 0.5 mg/mL protein concentration with the addition of 100 µM PLP. Spectra are the average of three independent samples and the tautomeric ratio ($CD_{335nm}/CD_{420\ nm}$) is reported as mean±SD. Color code is at follows: WT (blue), R347Q (cyan), L353P (red): the holo species are represented as straight lines, the apo species as dashed lines. **E)** Absorbance spectra of 1 mg/mL WT, R347Q, L353P after the addition of 2 mM DME. Incubations were performed in 100 mM potassium phosphate, pH 7.4 at 25 ◦C for 30 min. Dashed lines represents the time zero (internal aldimine) while solid lines the time dependent spectra recorded in the presence of DME. The maximum of absorbance of the external aldimine species is indicated, and the arrows show the absorbance band at 500 nm.

.

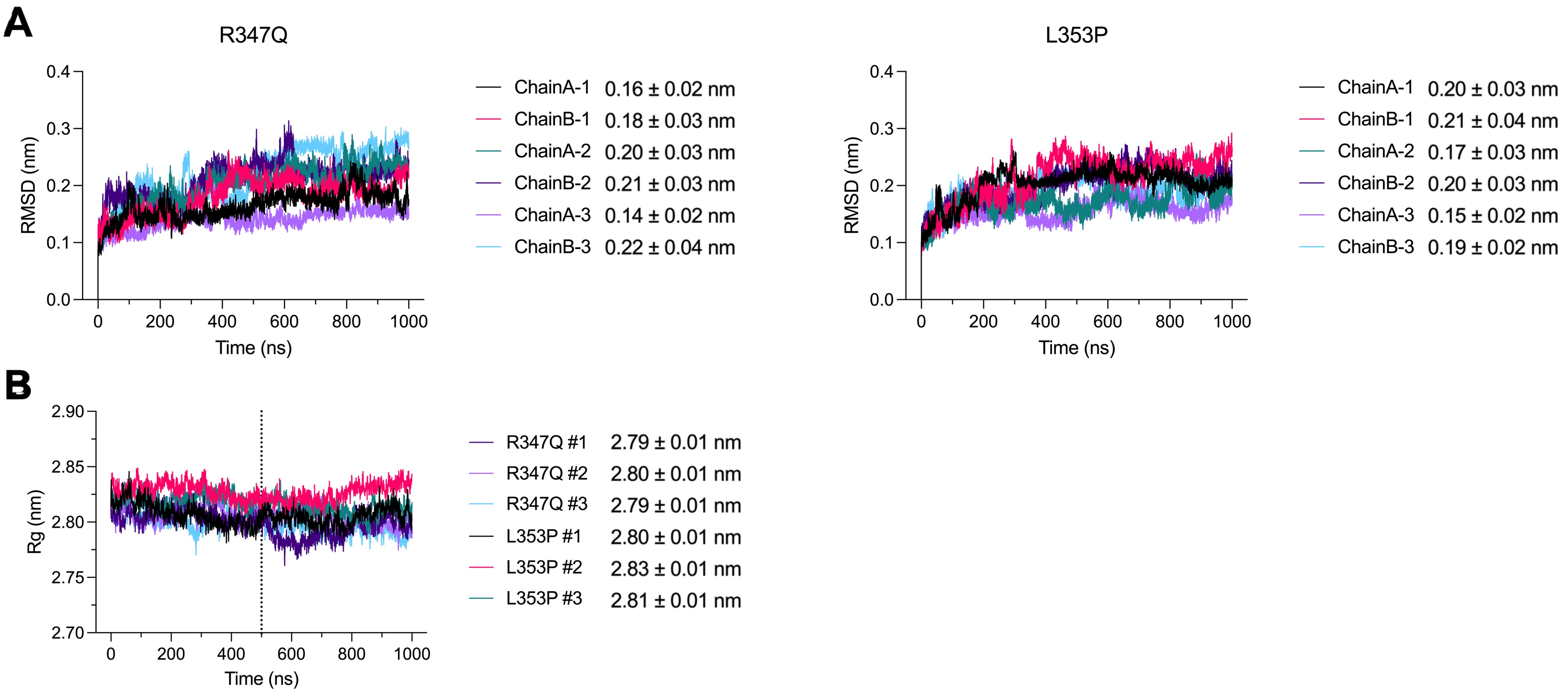

Figure S3. RMSD and gyration radius (Rg) determination by MD simulations of R347Q and L353P. A) Backbone RMSD as function of time for representative trajectories of each replica of simulation of single subunit chain. The reached plateau is reported as mean±SD. **B)** Rg calculated from each replica. The mean ± SD after convergence is reported.

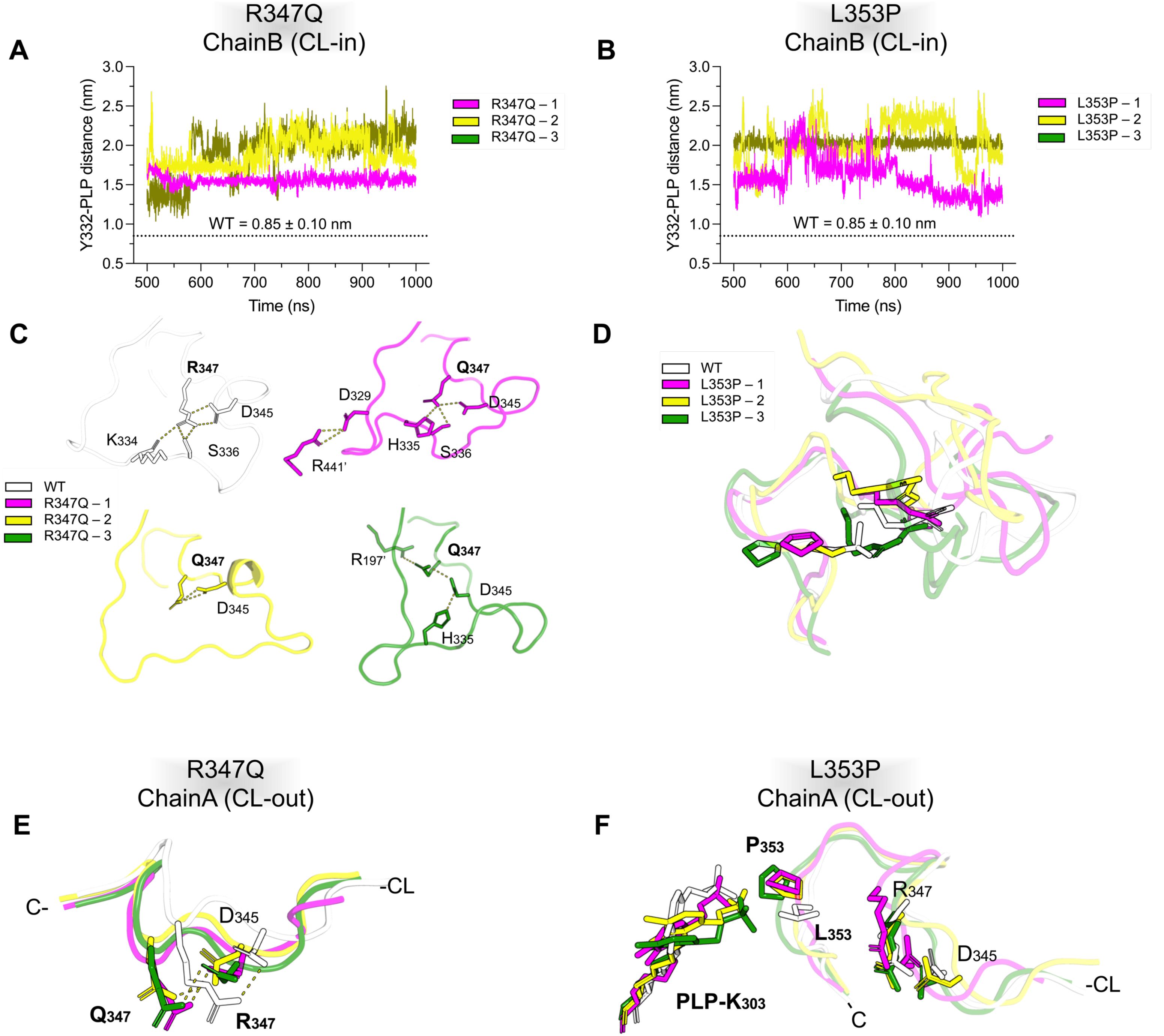

Fig. S4. Tyr332-PLP distance, CL-in and CL-out conformations from MD simulations of R327Q and L353P AADC variants. A-B) Distance (nm) between the hydroxyl group of the catalytic Tyr332 and the C4' of PLP along the last 500 ns of the three MD simulation replicas (different colors) of R347Q and L353P variants, respectively. The reference average distance between Tyr332 and PLP of the WT MD is reported for reference (dotted line). **C)** Comparison between the representative clusters of the CL-in conformation for R347Q MD simulations. Residues involved in interactions with Gln347 are shown, together with the WT similar conformation (white). The prime denotes residues belonging to the neighboring subunit (chain A). **D)** Comparison between the representative clusters of the CL-in conformation for L353P MD simulations. WT similar conformation (white) is reported for reference. **E)** loop3 CL-out conformation (residues 341-351) showing Gln347 interaction with Asp345 of the three replica and the WT similar conformation (white). **F)** loop 3 CL-Out conformation in the three replica of L353P MD simulations in comparison to WT (white). Figures were rendered with PyMol 2.3.4 (Schrödinger).

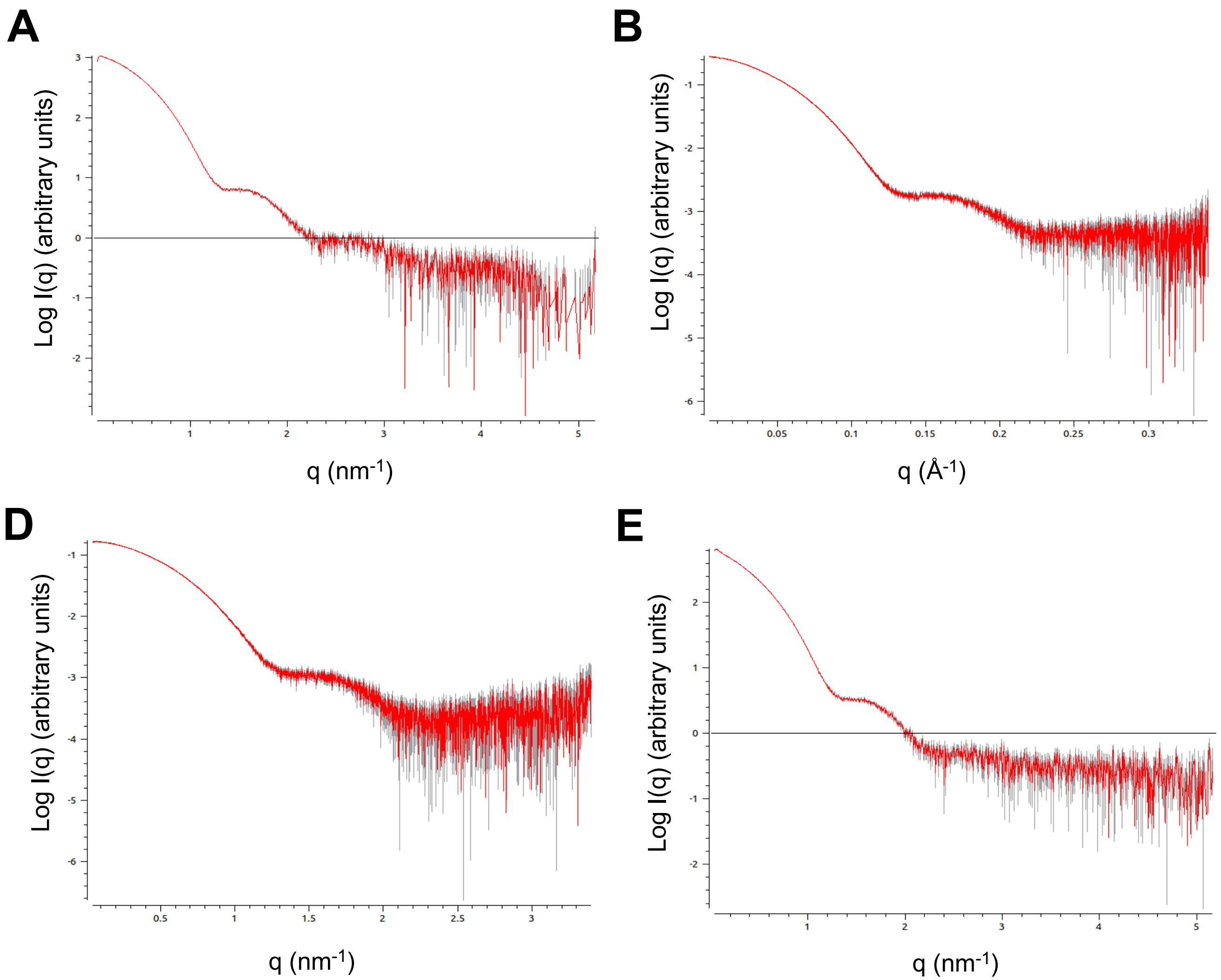

Fig. S5. SAXS profiles. A) WT in batch mode, **B)** WT in SEC-SAXS mode, **C)** L353P in batch mode, **D)** L353P in SEC-SAXS model, **E)** R347Q in batch mode.

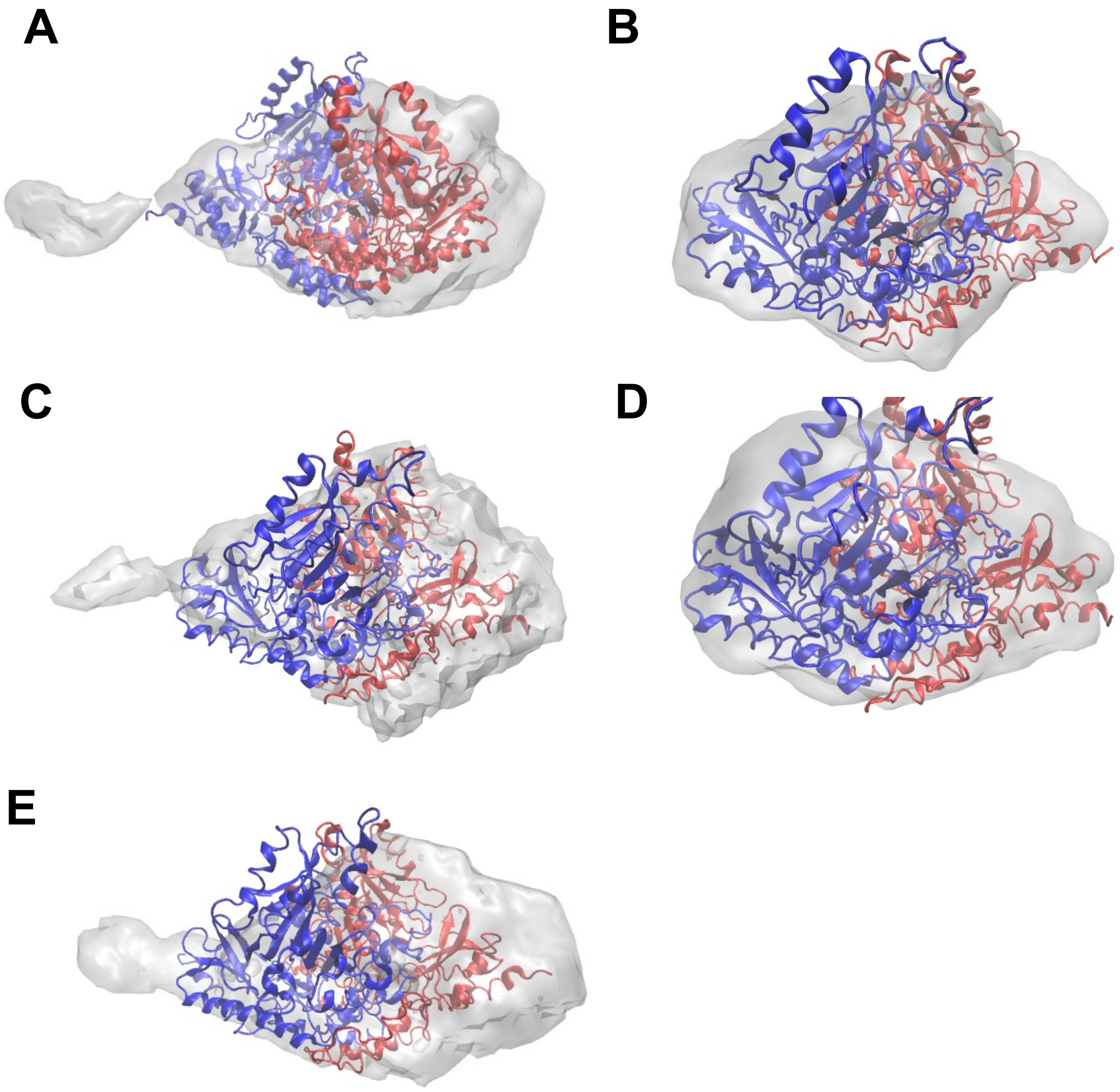

Fig. S6. ***Ab initio*** **SAXS molecular envelopes superposed to the atomistic models in best agreement with SAXS data. A)** WT in batch mode, **B)** WT in SEC-SAXS mode, **C)** L353P in batch mode, **D)** L353P in SEC-SAXS model, **E)** R347Q in batch mode. Chain A and Chain B are colored red and blue, respectively. Figures have been generated by using the program VMD [62].

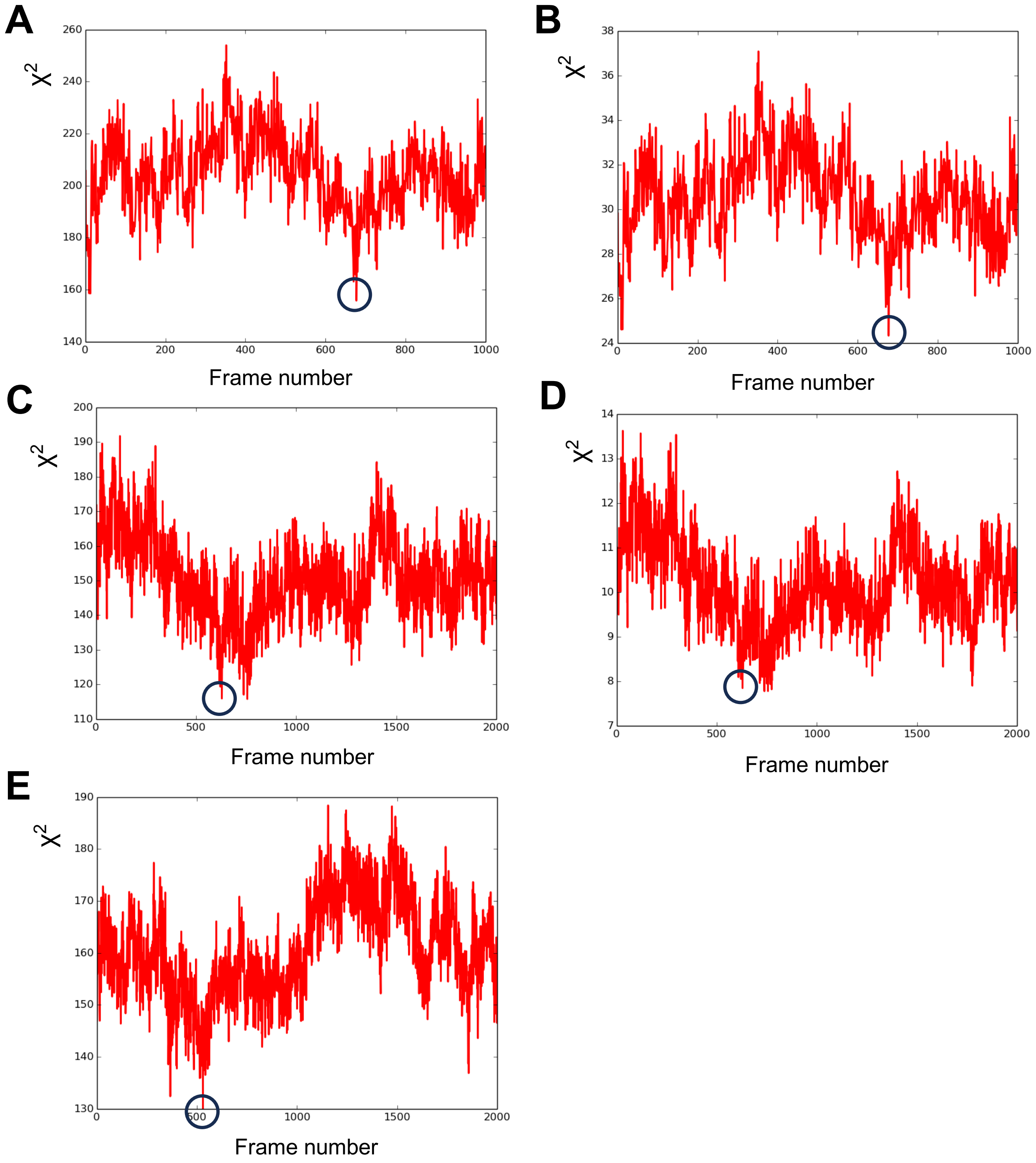

Fig. S7. χ^2 values obtained by fitting each frame of the MD simulations against SAXS profiles. A) WT, batch SAXS data; **B)** WT, SEC-SAXS data; **C)** L353P batch SAXS data; **D)** L353P, SEC-SAXS data; **E)** R347Q, batch SAXS data. The frames for which the minimum χ^2 value is reached are highlighted with a circle. For L353P and R347Q three MD replica have been screening, here those where the minimum χ^2 value is reached are reported.

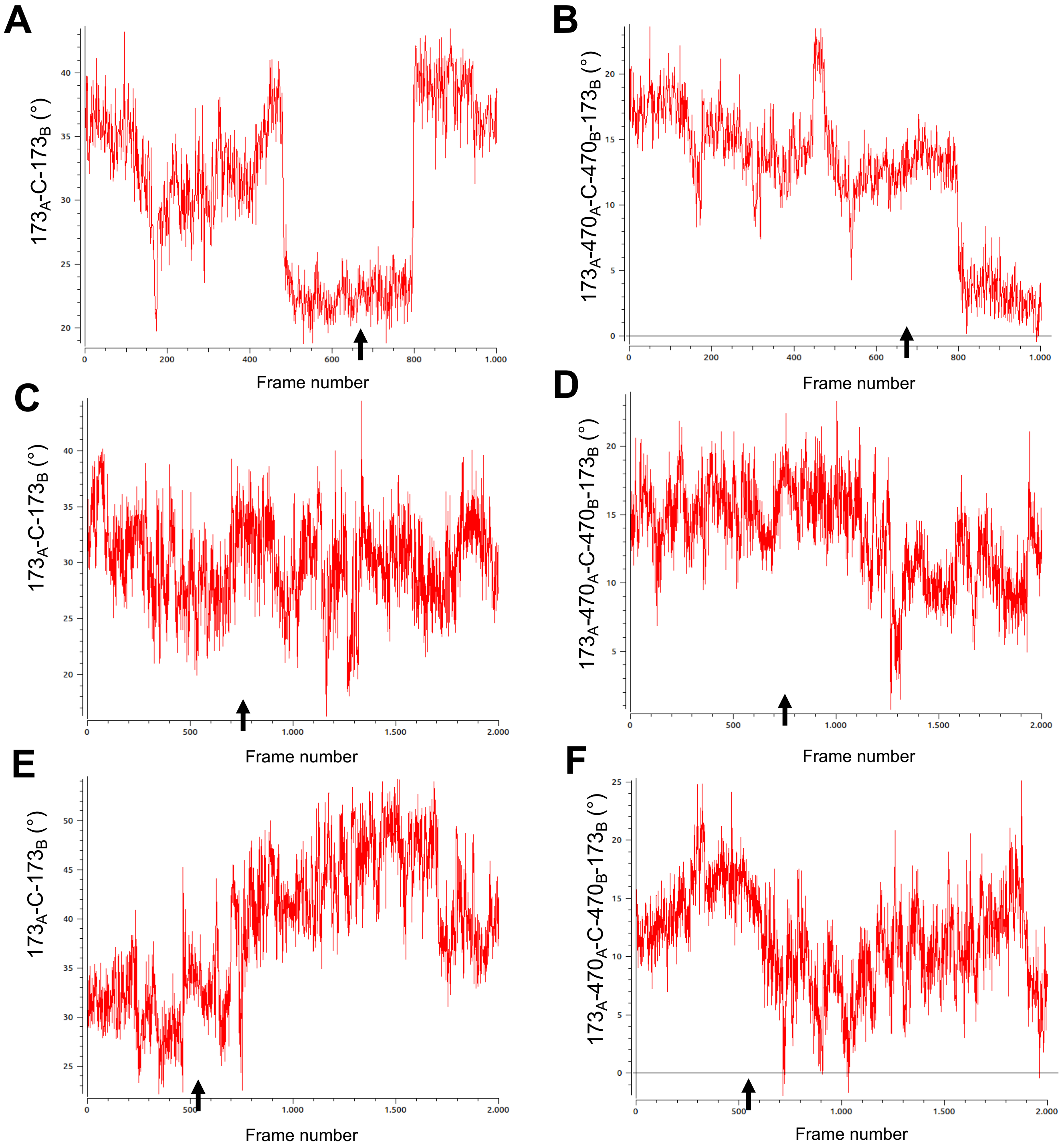

Fig. S8. Values of angular variables related to the hinge motion of chain A and chain B of the AADC dimer calculated for each frame of the MD simulations. Angle formed by Glu173 of the two chains (173_A and 173_B) with the center of mass of the dimer (C) for **A)** WT, **C)** L353P and **E)** R347Q. Dihedral angle formed by Glu173 and Leu470 of the two chains for **B)** WT, **D)** L353Po and **F)** R347Q. The frames for which the minimum χ^2 value is reached are highlighted with an arrow. For L353P and R347Q three MD replica have been screened, here those where the minimum χ^2 value is reached are reported.

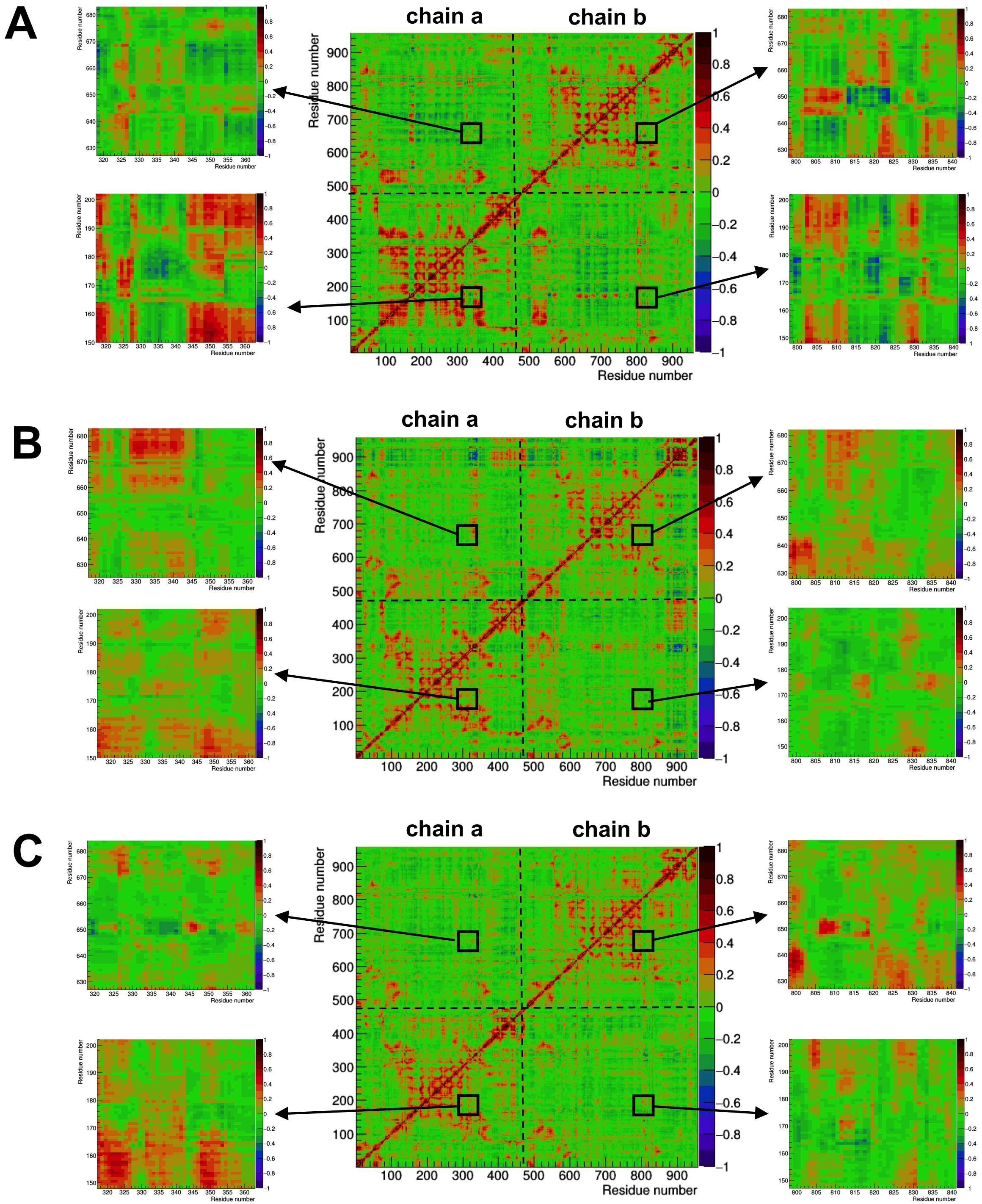

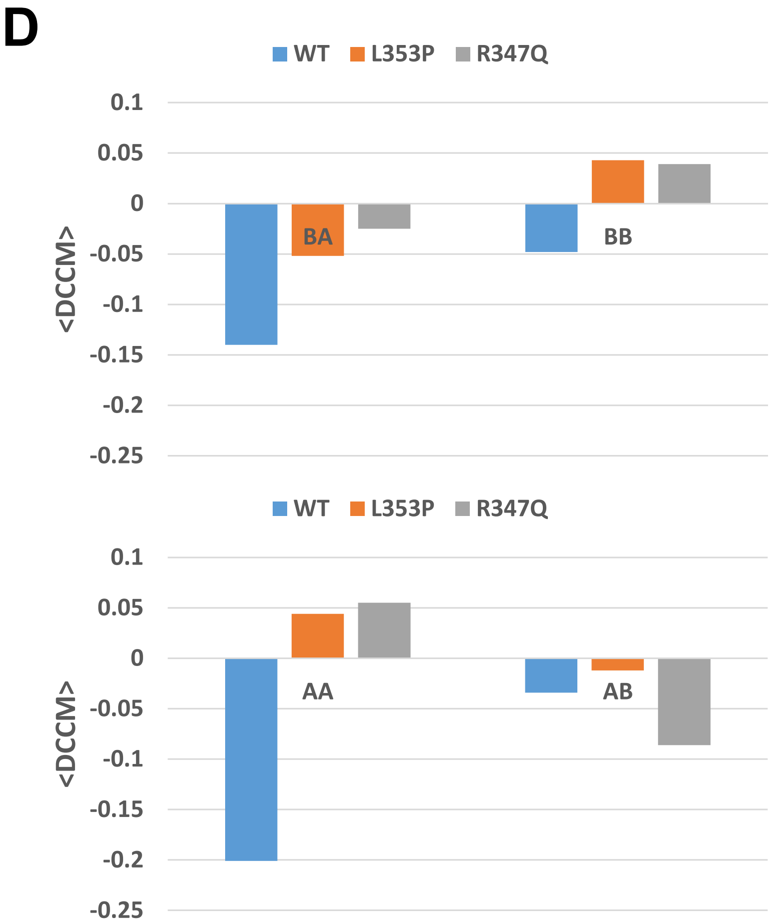

Fig. S9. Dynamical Cross-Correlation Matrix (DCCM) of the AADC dimer calculated for each frame of the MD simulations for **A)** WT, **B)** L353P variant and **C)** R347Q variant. Insets zoom on regions related to the interaction between loop3 and helix 6. **D)** Average DCCM values calculated in the region of interaction between the more exposed residues of loop3 (330-345) with residues of α-helix 6 (170-180) for chains A and B and for the three systems. For L353P and R347Q variants the MD replica containing the frame that best fits SAXS data have been considered.
